# Structure−Dynamics−Property Relationship Reveals the Origin of Porous Integrity in Imine-Linked Covalent Organic Frameworks

**DOI:** 10.1021/jacs.6c14616

**Published:** 2026-09-30

**Authors:** William B. Stoll, Johanna Kölbel, Alonso Acosta Vera, Sameeha Sultana, William Archacki, Khai-Nghi Truong, Irin E. Aby, Brandon R. Barnett, Luca Catalano, Agnes E. Thorarinsdottir, Michael T. Ruggiero

**Affiliations:** † Department of Chemistry, 6927 University of Rochester, Rochester, New York 14627, United States; § Rigaku Europe SE, Hugenottenallee 167, 63263 Neu-Isenburg, Germany; ∥ Dynamic Molecular Materials Laboratory, Department of Life Sciences, 9306 University of Modena and Reggio Emilia, Modena 41125, Italy; ⊥ Department of Chemical Engineering, University of Rochester, Rochester, New York 14627, United States; # CMAC, Strathclyde Institute of Pharmacy and Biomedical Sciences, University of Strathclyde, Glasgow G4 0RE, U.K.; △ Department of Chemistry, Yale University, New Haven, Connecticut 06511, United States

## Abstract

Molecular flexibility encoded in low-frequency lattice dynamics is increasingly recognized as a key determinant of the properties of porous materials, particularly metal–organic frameworks, but remains largely unexplored in covalent organic frameworks (COFs). Here, we establish a structure–dynamics–property relationship for chemically related COFs by combining three-dimensional electron diffraction, low-frequency vibrational spectroscopy, and first-principles calculations. Using 3D electron diffraction, we determined the crystal structure of the collapsed reduced framework COF-300-AR, revealing a bent linker conformation. Terahertz and low-frequency Raman spectroscopy, together with solid-state density functional theory and local mode analysis, resolve the lattice dynamics of contracted COF-300-H_2_O, COF-320A, and collapsed COF-300-AR and quantify linker flexibility through imine/amine torsions. Normal-mode decomposition and torsional potential energy surfaces show that these coordinates are comparatively stiff in the imine-based frameworks, preserving near-planar linker conformations that support guest-responsive structures. In contrast, COF-300-AR exhibits a shallower, multiwell torsional potential that enables bent linker conformations, favoring collapsed packing. Gas-phase local-mode analysis reproduces this relative trend in torsional softness, suggesting that linker flexibility can be screened before a crystal structure is available. The negligible gas uptake of COF-300-AR is therefore linked to the coupling of local amine flexibility with packing constraints in the interpenetrated framework. More broadly, these results demonstrate how low-frequency dynamics can identify molecular coordinates governing the structural integrity and porosity in flexible COFs.

## Graphical abstract

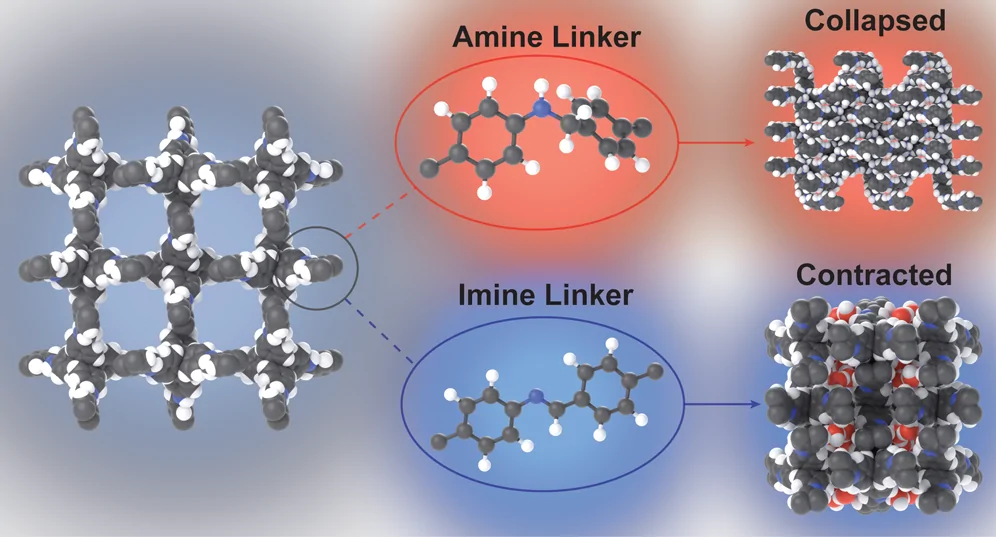

## Introduction

Molecular flexibility, defined here as the energetic penalty associated with torsions or rotations within molecular conformers, is crucial for functions ranging from protein folding and mechanical response, to the bulk behavior of porous molecular materials.^1–6^ This definition is distinct from bulk elastic flexibility, where an entire crystal bends or deforms reversibly under an applied mechanical force as observed in flexible crystals,^7^ and from the broader framework-level flexibility of porous materials, where pores can open, close, or change shape in response to guests, pressure, or temperature.^8^ Instead, it focuses on the conformational energy landscape of molecular building units, which ultimately underpins many material properties. For example, in metal–organic frameworks (MOFs), linker flexibility gives rise to collective motions such as pore breathing, linker librations, and layer sliding, which in turn govern gas adsorption and diffusion, and—in the case of so-called “flexible MOFs”—permit large-scale structural transformations of the entire framework.^9–12^

Over the past decade, terahertz and low-frequency Raman spectroscopy, in combination with first-principles simulations, have become powerful tools for studying low-frequency motions associated with this molecular flexibility in porous materials such as MOFs, zeolites, and clathrates.^10,11,13–15^ In this low-frequency region, vibrations typically involve collective motions, such as linker torsions, hindered rotations, framework shear, and pore-breathing distortions, rather than isolated bond stretches or bends.^16,17^ These motions occur at low frequencies because they sample shallow portions of the potential energy surface where the restoring force from weak intermolecular interactions or torsions is small and because they involve the motion of relatively large molecular or framework fragments. When combined with first-principles simulations, terahertz spectroscopy directly links the energy landscape to the soft coordinates that define the molecular flexibility of the material.^9,10^

In contrast, the low-frequency dynamics of covalent organic frameworks (COFs) remain unexplored, despite their structural similarity to MOFs and their growing prominence in catalysis and charge transport.^18–22^ Recent works have investigated the prototypical COF-300, its reduced analogue COF-300-AR, and the extended atropisomer COF-320, highlighting their guest-dependent structural responses.^23–28^ Yet, there are still very few studies that resolve the low-frequency intermolecular vibrational motions and specific intramolecular conformational changes that underpin the flexible response of these and other COFs. This gap in knowledge is striking given the enhanced robustness and chemical stability of COFs toward guest inclusion,^29^ rendering them increasingly important in chemical recognition,^30,31^ electrocatalysis,^18,21^ and charge transport.^20,22^

Here, we address this knowledge gap by establishing explicit structure−dynamics–property relationships for chemically related COFs and by quantifying flexibility as the energetic penalty for rotation around imine or amine linker torsions. We use three-dimensional electron diffraction (3D-ED)^32,33^ to determine the crystal structure of a collapsed COF-300-AR phase and to compare its linker geometry with imine-linked COF-300-H_2_O and COF-320A. We then combine terahertz and low-frequency Raman spectroscopy with solid-state density functional theory (ss-DFT) and local mode analysis to elucidate the low-frequency lattice dynamics of hydrated contracted COF-300-H_2_O, COF-320A, and collapsed COF-300-AR, focusing on modes that involve the imine or amine linkers. By deconvoluting the normal modes into internal coordinates and extracting effective force constants for the relevant linker dihedral angles, complemented by potential energy scans along these torsions, we quantify the rotational softness of the linkers across the three frameworks. We show that imine-linked COF-300-H_2_O and COF-320A retain comparatively stiff, near-planar linker conformations, while postsynthetic reduction of COF-300-H_2_O to COF-300-AR produces a softer C–N torsional landscape that allows bent linker conformations and favors collapsed packing. In COF-320A, an additional phenyl torsion in the linker introduces another rotational degree of freedom while preserving the rigid imine-bridged fragment. By directly linking the experimentally determined structures, the dynamics of specific low-frequency modes, and the resulting energetic flexibility, our results reveal how structural integrity and adaptability arise in these COFs and demonstrate a general strategy for using low-frequency vibrational spectroscopy and first-principles simulations to interrogate flexible COFs.

## Results and Discussion

### Synthesis and Structures

All COF samples were synthesized using modified literature procedures with detailed experimental conditions found in the Supporting Information (Figures S1–S3 and Table S1).^28,34,35^ For COF-300-H_2_O and COF-320A, the experimental powder X-ray diffraction (PXRD) patterns (Figures S4 and S5) and Fourier transform infrared (FTIR) spectra (Figures S6 and S7) were in close agreement with previous reports,^28,34^ confirming their crystallinity and molecular composition. 3D-ED was performed on COF-300-H_2_O at 175 K in ultrahigh vacuum, confirming the hydrated contracted dia-c7 phase in accordance with previous reports,^25,26,36–41^ with a node-to-node length of 18.45 Å and imine-bridged dihedral angle of φ_1_ = 167.35° (Figure 1a,b and Table S5). One unit cell contains four water molecules, forming strong hydrogen bonding interactions with distances of 2.09 and 1.79 Å for the N···H–O and O–H···O–H contacts, respectively, which prompt a guest-induced pore contraction that results in the contracted-phase of the framework.^26^ COF-300 frameworks are known to access different interpenetration and conformational states depending on synthetic conditions and guest inclusion.^42^ This sample is therefore assigned as the hydrated contracted (but not fully closed) phase COF-300-H_2_O in which the framework retains a porous architecture but the accessible channel volume is occupied by strongly bound water molecules. Nitrogen adsorption isotherm measurements for COF-300-H_2_O at 77 K revealed negligible uptake of N_2_ (Figure S8).^43^ The stability of the hydrated contracted phase under the thermal and vacuum treatments used in the subsequent measurements was further evaluated by thermal gravimetric analysis (TGA). Following equilibration at 50 °C, no distinct additional low-temperature mass-loss event is observed over the temperature range relevant to activation (Figure S9).

**Figure 1:**
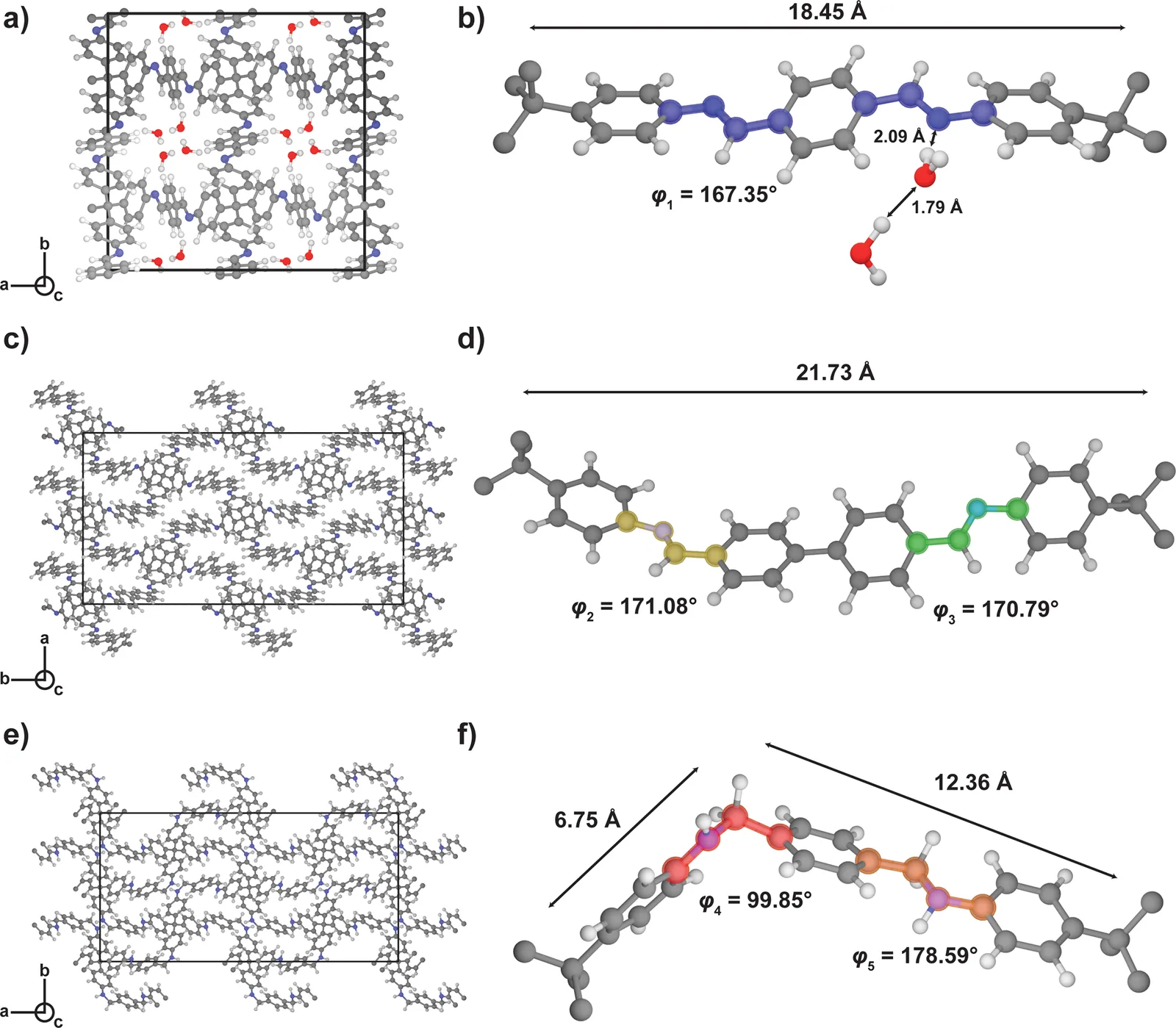
Structure of COF-300-H_2_O (a, b), COF-320A (c, d), and COF-300-AR (e, f). Unit cell packing (a, c, e) and linker geometry (b, d, f). Each of the linker geometries is labeled with node-to-node lengths, and the torsional angle of the bridging imine/amine fragment in the linker is highlighted and labeled.

Consistently, the vibrational spectra before and after vacuum treatment (Figure S10) show close agreement with the periodic calculations of hydrated COF-300-H_2_O, supporting the retention of the hydrated contracted framework state under the conditions studied and confirming no detectable transformation to a different framework phase.

For its extended analogue COF-320A, the collapsed-pore structure stems from atropisomerism, wherein the imine bonds are oriented toward the same side of the biphenyl linker with two distinct dihedral angles of φ_2_ = 171.08° and φ_3_ = 170.79°, caused by the structural torsion between the phenyl rings of the aldehyde moiety (Figure 1c,d). In the case of COF-320A, a type IV isotherm was observed upon N_2_ adsorption at 77 K (Figure S11), confirming the previously reported pressure-induced structural transition in the framework.^27^

A postsynthetic modification of COF-300-H_2_O was carried out by reducing the imine bonds to obtain COF-300-AR, as monitored by FTIR spectroscopy (Figures S12 and S13).^24^ Note that COF-300-AR is chemically distinct from COF-300-H_2_O and cannot be obtained by solvent exchange or activation. PXRD analysis of COF-300-AR indicates the formation of a crystalline material with a significantly different structure from that of COF-300-H_2_O (Figures S14 and S15). 3D-ED analysis of a crystalline powder of COF-300-AR (Figures S16–S19 and Tables S2-S4) reveals that the structure crystallizes in the orthorhombic *Fdd*2 space group with unit cell parameters of *a* = 40.75 Å, *b* = 20.83 Å, and *c* = 7.05 Å at 175 K (Figure 1e). The linker exhibits two symmetry-independent dihedral angles between the amine groups and the phenyl ring with values of φ_4_ = 99.85° and φ_5_ = 178.59° and lengths of 6.75 and 12.36 Å for the two respective portions of the fragment (Figure 1f). Both dihedral angles correspond to torsional coordinates of the reduced amine-linked backbone, but they adopt different geometries in the crystalline framework.

The characteristic bent linker conformation enables the collapsed, non-porous form, which is reflected in negligible N_2_ uptake at 77 K (Figure S20). Notably, a previously reported collapsed-pore phase was solved in the *I*4_1_/*a* space group instead of the *Fdd*2 space group reported here.^44^ The previously reported structure exhibits two symmetry related amine bridges per linker with dihedral angles of 99.90°. In comparison to the COF-300-AR structure presented in this paper, both bridges form a bent conformation, rather than only one. This structural difference may reflect the sensitivity of the reduced framework to synthesis, which can favor distinct packing arrangements accessible on the relatively shallow C–N torsional energy landscape. This comparison highlights how the molecular flexibility gained from chemical reduction can permit multiple stable forms and the observation of strongly bent C–N conformations in both structures is consistent with the ability of the reduced linker to access a broader range of conformations than the corresponding parent imine-linked framework.

### Low-Frequency Vibrational Spectroscopy

To elucidate the low-frequency dynamics of the flexible frameworks, samples were prepared and measured using a combination of terahertz time-domain spectroscopy (THz-TDS) and Raman spectroscopy according to previously described procedures.^45^ The temperature-dependent terahertz spectra of COF-300-H_2_O, COF-320A, and the collapsed-pore phase of COF-300-AR, respectively, are presented in Figure 2, and the low-frequency Raman spectra for COF-300-H_2_O and COF-300-AR are presented in Figure 3 (additional spectra are shown in Figures S21–S25). Note that the COF-320A framework burned at the laser wavelength of 785 nm used in the Raman experiments and was therefore only characterized with THz-TDS.

**Figure 2:**
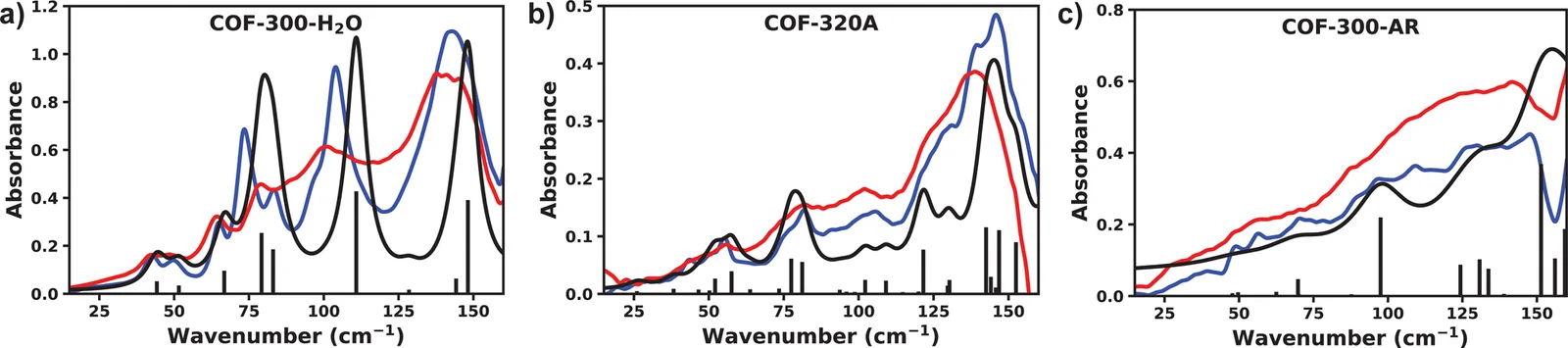
Low-frequency vibrational spectra of COF-300-H_2_O (a), COF-320A (b), and collapsed COF-300-AR (c) and comparison to calculated vibrational spectra (black). Terahertz spectra measured at 78 and 300 K are displayed in blue and red, respectively.

**Figure 3:**
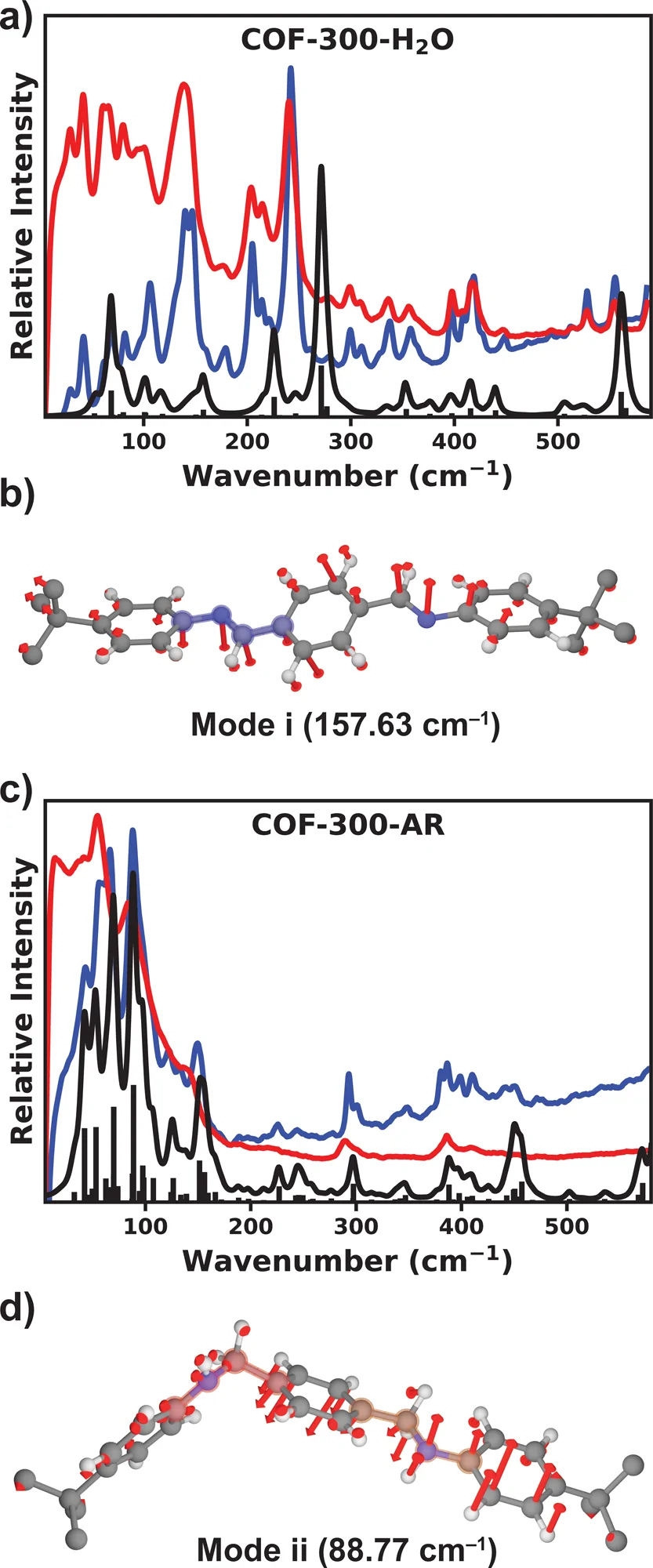
Low-frequency Raman spectra of COF-300-H_2_O (a) and COF-300-AR (c), as well as comparison to calculated vibrational spectra (black). Raman spectra measured at 78 and 300 K are displayed in blue and red, respectively. Visualization of representative vibrational coordinates that modulate the linker dihedral angles in COF-300-H_2_O (b, mode i) and in COF-300-AR (d, mode ii).

For the three systems, the terahertz spectra show multiple pronounced peaks that red-shift and broaden with increasing temperature. This is a typical signature observed in low-frequency vibrational spectroscopy experiments and signifies the anharmonic softening of the mode potential as temperature is elevated.^46,47^ The increase in background intensity at higher temperatures is attributed to dynamic disorder and increased molecular mobility.^48^ For COF-300-H_2_O, the large dipole moment of the water guest molecules interacting with the imine functional groups result in intense peaks representative of characteristic vibrations associated with the intermolecular hydrogen bonding inside the pores (Figure 2a).^16^ In contrast, spectral features for COF-300-AR are broader and only become apparent at low temperatures (Figure 2c). The experimental Raman spectra of the two systems show clear temperature- and guest-dependent differences in the low-frequency vibrational response (Figure 3). COF-300-H_2_O displays a broad distribution of Raman-active modes across the measured range with the 78 K spectrum showing sharper features than the 300 K spectrum. In contrast, COF-300-AR is dominated by a dense set of low-frequency modes below 150 cm^–1^. While it is common to assign vibrations in the infrared region of the electromagnetic spectrum with frequency tables, such information does not exist for the terahertz region.^12^ To assign these modes, fully-periodic ss-DFT calculations were performed with theoretical methodologies described in the SI.

Vibrational spectroscopy provides direct access to the curvature of the potential energy surface along specific nuclear coordinates with lower-frequency modes corresponding to softer, more easily deformed directions.^12^ Terahertz vibrations are a result of weak interactions, which often originate along torsional and/or intermolecular coordinates. Thus, by comparing the vibrational frequency for a particular coordinate across series of frameworks, in this case, the torsional linker coordinates, it is possible to obtain an indication of the strength of that particular coordinate’s interaction. Analysis of both the calculated frequency (i.e., the strength of the interaction) and eigenvector displacement pattern of terahertz modes hence allows the softest framework coordinates to be identified.

The mode assignments are based on the calculated normal-mode eigenvectors from ss-DFT simulations. Each eigenvector describes the direction and relative magnitude of the displacement of every atom in the periodic framework. Modes were therefore classified according to their dominant displacement pattern, including channel breathing, framework shear, and linker rotations involving changes in the imine or amine dihedral angles. These assignments are illustrated by displacement-arrow figures in the SI (Figure S26) and by the animated vibrational modes provided in the SI.

Local mode analysis provides an additional quantitative way to characterize collective normal modes by projecting them onto internal coordinates, such as bond lengths, bond angles, and dihedral torsions. Such an analysis allows the identification of which linker torsions contribute most strongly to the discussed low-frequency modes.

For example, in COF-300-H_2_O, mode i corresponds to the prominent Raman and terahertz bands located near 150 cm^–1^ (Figure 3b). The corresponding eigenvectors show the breathing of the channel, where imine linkers rotate around the φ_1_ dihedral angle, resulting in a cooperative mode that increases the channel cross section.

In COF-300-AR, mode ii corresponds to a prominent Raman band experimentally observed near 90 cm^–1^, where the eigenvectors map out the rotation of the amine linker (Figure 3d). Here, the collapsed interpenetrated structure places strong steric constraints on pore expansion, and no mode with dominant pore-breathing character is identified among the calculated harmonic Γ-point vibrations.

As many of the low-frequency modes involve conformational torsions of the linkers, an accurate reproduction of the spectra by theoretical methods implies that the energies related to conformational flexibility are well-modeled.^12^ The ss-DFT frequency calculations for each system result in excellent agreement between theory and the experiments (Figures 2 and 3), providing confidence in the modeled energy landscape.

However, many of the terahertz modes are deeply convoluted, and in the case of COF-300-H_2_O, they also involve the motion of guest molecules. Thus, the contributions of each internal coordinate (i.e., bond lengths, angles, and dihedral torsions) are deconvoluted to the overall energy using local mode analysis.^49,50^ The resulting values describing the torsions of the imine/amine-bridged fragment, which are most relevant to molecular flexibility in the frameworks, are presented in Figure 4.

**Figure 4:**
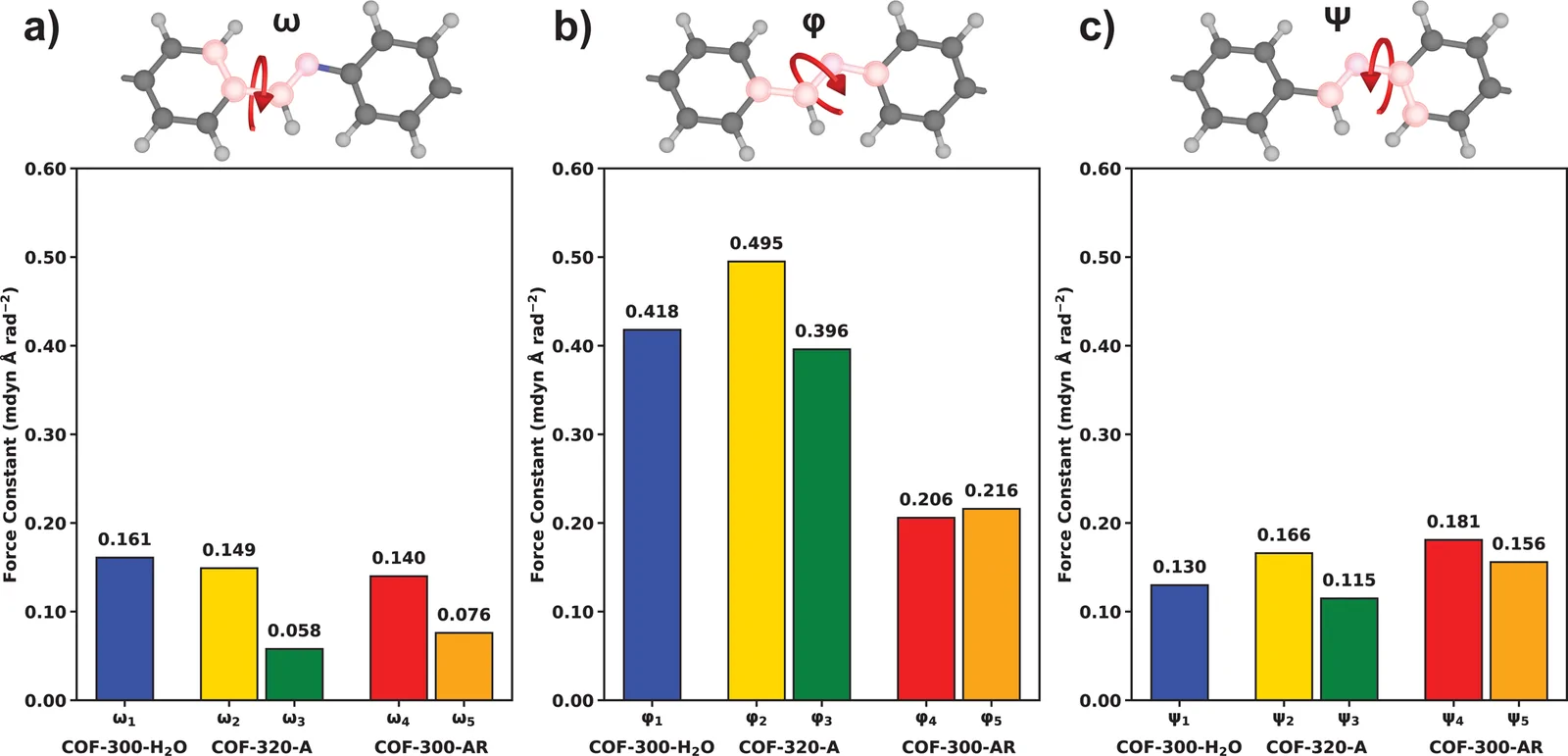
Calculated torsional force constants associated with the internal coordinates describing the imine/amine-bridged fragment in the structural backbone of COF-300-H_2_O, COF-320A, and collapsed-pore COF-300-AR. Quantitative analysis of the molecular flexibility describing the pedal-type motion (a, c), and the dihedral torsion of the imine/amine bonds (b). The dihedral angles are labeled with ω, φ, and ψ for a, b, and c, respectively.

In all the frameworks, the softest rotational potential arises from the imine/amine group pedal-type motion, ranging from 0.130 to 0.161 mdyn Å rad^–2^ in COF-300-H_2_O, 0.058 to 0.166 mdyn Å rad^–2^ in COF-320A, and 0.076 to 0.181 mdyn Å rad^–2^ in COF-300-AR (Figure 4a,c). The calculated force constants for the dihedral torsions of the imine-bridged fragment in COF-300-H_2_O and COF-320A possess similar values of 0.418 mdyn Å rad^–2^ (for φ_1_) and 0.495 mdyn Å rad^–2^ and 0.396 mdyn Å rad^–2^ (for φ_2_ and φ_3_, respectively) (Figure 4b). In contrast, in the case of COF-300-AR, the force constants associated with the rotation around the amine-bridged fragment equal 0.206 and 0.216 mdyn Å rad^–2^ (for φ_4_ and φ_5_, respectively) (Figure 4b), indicating that the linker torsion is roughly twice as stiff in the imine-based structures. Consequently, the relatively lower torsional force constant (for φ_4_ and φ_5_) is permissive of a bent conformation in the linker, which leads to the nonporous COF-300-AR structure. For COF-320A, the torsional force constant between the phenyl rings is 0.133 mdyn Å rad^–2^, providing an additional rotational degree of freedom to the system. This additional rotatable bond increases the conformational flexibility of the linker, as the force constant is comparable to that of the pedal-type motions (Figures 4a,c and S27).

While local mode analysis in the solid state requires previous knowledge of the crystal structure, the overall methodology can be applied *a priori* using exclusively the linker geometry in the gas phase. To test the validity of such simulations, vibrational frequency calculations were performed along with local-mode analysis on each linker fragment. The absolute values of the local torsional force constants in the gas phase are consistently lower in comparison to the values calculated using ss-DFT due to steric hindrance and intermolecular interactions imposed by the framework. Still, the overall trend is conserved; that is, the force constant corresponding to amine bond rotation is substantially smaller than the corresponding value for rotation around the imine bond (Figure S27).

The local torsional force constants reported here quantify the energetic resistance associated with a specific linker coordinate, whereas framework-level flexibility reflects the cooperative response of the periodic structure.^23^ This response depends on the local linker flexibility, but is additionally influenced by topology, interpenetration, crystal packing, and host–guest interactions.

For example, the torsional softness of the amine linkage controls the range of locally accessible linker conformations, whereas the interpenetrated dia-c7 packing governs how these conformations are accommodated within the periodic framework. In COF-300-AR, the combination of a soft amine torsion and the steric constraints imposed by the interpenetrated framework enables bent linker geometries compatible with the collapsed pore structure. In the absence of steric restrictions, rotation around the σ-bond would be expected to have a comparatively low activation energy barrier, providing insight into the energy landscape that underlies irreversible structural collapse after postsynthetic modification of the parent imine-linked framework. The relationship between these local energetic coordinates and the structural pathway connecting open and collapsed framework states provides an important direction for future study.

### Torsional Energy Landscapes

To fully comprehend why the linker rotations are significantly softer in COF-300-AR than in both imine-based frameworks COF-300-H_2_O and COF-320A, the underlying torsional potentials were examined. One-dimensional scans of the potential energy surface were performed as a function of the φ_1_, φ_3_, and φ_4_ torsion angles in the gas phase using Gaussian16. A representative molecular fragment of the local linker environment was used for each framework (Figure S28), allowing the geometry to fully relax at each torsional step (Figure 5). The resulting torsional profiles are different between the two imine fragments and the reduced derivative. The rotational barrier is approximately an order of magnitude larger for both COF-300-H_2_O and COF-320A than for COF-300-AR, consistent with the larger torsional force constants determined previously (Figure 4b).

**Figure 5:**
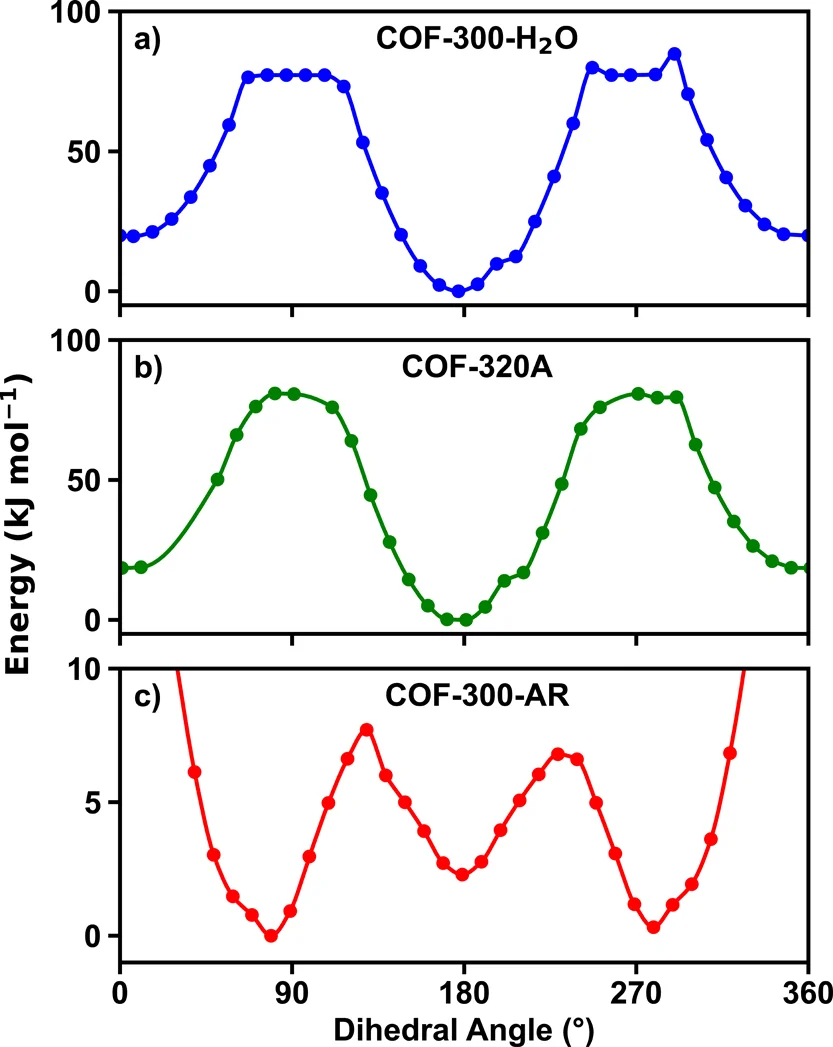
1D relaxed potential energy surface scan of the dihedral angle for linker fragments in COF-300-H_2_O (φ_1_) (a), COF-320A (φ_3_) (b), and collapsed COF-300-AR (φ_4_) (c).

In COF-300-H_2_O and COF-320A, the linkers exhibit a deep, nearly harmonic well centered at 180°, corresponding to the experimentally observed planar conformation between the imine groups and the adjacent phenyl rings (φ_1_ = 167.35° for COF-300-H_2_O and φ_2_ = 171.08° and φ_3_ = 170.79° for COF-320A). The two potential energy wells fall 180° apart, closely resembling *cis* and *trans* isomers (Figure 5a,b). Here, the expanded backbone of COF-320A has little effect on the torsional potential. The rigidity of the dihedral torsions φ_1_, φ_2_, and φ_3_ arises from the π-bonding between the carbon and nitrogen atoms in the imine groups, reflected in the observed large activation energy barrier (see also Figure S29 for an illustration of the C–H···π interaction).^51^

For COF-300-H_2_O, including a water molecule in the scan provides insight into how the guest modulates the torsional potential. Figure S30 illustrates the difference between the rotational energy landscapes with and without the 2.09 Å hydrogen-bonding contact (Figure 1b). The shape of the potential near the minimum is similar between the two scans as the energy is largely dominated by strain of the imine π-bond. However, at larger dihedral angles, the presence of the water molecules induces a sharp increase in energy due to steric hindrance between the phenyl group and water molecules. Thus, the guest restricts the torsional motions of the imine-based linkers. This restriction is also paired with a strong hydrogen-bonding network; the solid-state local mode analysis yields a force constant of 0.045 mdyn Å rad–2 for the 2.09 Å water···framework interaction and a force constant of 0.093 mdyn Å rad–2 for the 1.79 Å water···water interaction (N···H–O and O–H···O–H contacts, respectively). Thus, the guest···guest interaction is about twice as strong as the guest···framework interaction. Together, these results indicate that guest inclusion stabilizes the pore both by damping torsional motion of the linker and by establishing a hydrogen-bonding network.

In the case of COF-300-AR, the torsional potentials are much flatter and display two global energy minima at dihedral angles of approximately 80° and 280° (Figure 5c). The bent linker geometry in COF-300-AR described by φ_4_ = 99.85° falls within the well centered at 80°. The slight discrepancy relative to the experimentally determined structure can be attributed to the backbone adopting a higher-energy conformation that is stabilized by an intermolecular 2.57 Å C–H···π interaction (Figure S29). The potential energy scan of COF-300-AR also displays a shallower energy minimum (which resembles a saddle point in the rigid structure) at 180°, corresponding to the planar conformation. This behavior reflects the greater conformational freedom of the reduced C–N bond, which can adopt both linear and bent geometries because it is less constrained by electronic conjugation.

## Conclusion

These results establish explicit structure–dynamics–property relationships for chemically related COFs by combining 3D-ED, low-frequency vibrational spectroscopy, and first-principles calculations. Specifically, the comparison between COF-300-H_2_O and COF-320A enabled us to probe the influence of guest inclusion, linker expansion, and additional phenyl torsion on low-frequency lattice dynamics. Comparing the results of the two imine-linked frameworks with the reduced framework COF-300-AR, which exhibits a collapsed structure with bent linker conformation, as revealed by 3D-ED, further allowed us to investigate the impact of linker conformation and rotational flexibility on lattice dynamics. Terahertz and low-frequency Raman spectroscopy, combined with solid-state DFT and local mode analysis, allowed us to resolve the low-frequency lattice dynamics of hydrated contracted COF-300-H_2_O, COF-320A, and collapsed COF-300-AR and to quantify linker flexibility in terms of C=N/C–N torsions. Deconvolution of the normal modes and torsional potential scans shows that the linker torsion is comparatively stiff in the imine-based frameworks, COF-300-H_2_O and COF-320A, and that the reduced framework has a shallow, multiwell torsional energy landscape around the C–N bond. These results illustrate that the C=N/C–N torsions that contribute most strongly to the linker-centered low-frequency modes are softer in collapsed COF-300-AR than in the imine-linked frameworks.

Thus, at the level of the local linker dihedral angle, the reduced framework possesses a more-permissive torsional potential. This enhanced local molecular flexibility allows the bent linker conformation to be stabilized, making the collapsed packing arrangement of the COF-300-AR accessible under the conditions studied here. In contrast, the imine-linked frameworks retain deeper torsional energy wells near the planar conformation, preserving the near-planar linker geometries.

The complementary gas-phase local-mode analysis further indicates that these trends are encoded in the linker fragments themselves. Although the absolute torsional force constants differ between the solid-state and gas-phase calculations, the relative trend is preserved with the amine-bridged torsion remaining substantially softer than the corresponding imine-bridged torsions.

Because the gas-phase analysis requires only knowledge of the linker geometry, it provides a potential route for evaluating linker conformational energy landscapes before a crystal structure is solved. This is particularly valuable for COFs, where obtaining large single crystals suitable for structure determination remains challenging and experimentally determined crystal structures are comparatively rare. Because the gas-phase calculations do not include guests or crystal-packing effects, they also help isolate the intrinsic rotational barrier of the linker fragment from host–guest and solid-state interactions. In this way, linker fragments could be screened for rigid (“peg-like”) or more conformationally flexible (“hinge-like”) behavior, providing guidance for the design of COFs with targeted structural responses in the absence of large single crystals before full structural characterization is available.

The negligible gas uptake of COF-300-AR can therefore be traced to the coupling between a locally soft amine linker and the packing constraints of the interpenetrated framework. In other words, the ability of the C–N bond to rotate and accommodate the bent linker conformation is what stabilizes the collapsed packing arrangement of COF-300-AR, whereas an analogous imine-linked framework cannot realize an equivalent collapsed structure without incurring a much higher energetic cost. More broadly, this work highlights that low-frequency vibrational spectroscopy, structure determination, and coordinate-resolved calculations can identify and characterize the linker distortions that govern pore opening, guest uptake, and structural integrity in flexible COFs,^52^ while gas-phase linker analysis provides a first step toward predictive screening of new framework building blocks for targeted applications. Building on this approach, future studies can directly examine how these local energetic coordinates participate in the cooperative structural transformations between open and collapsed frameworks during *in situ* spectroscopic experiments.

## Supplementary Material







## Notes

Mode visualizations are included as .xyz files in a compressed folder for COF-300-H_2_O, COF-320A, and COF-300-AR (ZIP)
Experimental and synthesis procedures; elemental analysis; PXRD, FTIR-ATR, N_2_ adsorption, TGA, 3D-ED, Raman and terahertz spectroscopy data; crystallographic data and refinement parameters; computational methods; normal-mode analysis, force constants, and torsional potential energy surfaces (PDF)
