# Supplementary material for: Structure−Dynamics−Property Relationship Reveals the Origin of Porous Integrity in Imine-Linked Covalent Organic Frameworks
Source: J Am Chem Soc. 2026 Sep 30;148(40):43682–90. doi: 10.1021/jacs.6c14616 (PMC13674272; doi:10.1021/jacs.6c14616)
Supplement: Supplementary file 1 [file ja6c14616_si_003.pdf]

Structure–Dynamics–Property Relationship Reveals the Origin of Porous Integrity in Imine-Linked Covalent Organic Frameworks

William B. Stoll,[†,‡] Johanna Kölbel,[†,‡] Alonso Acosta Vera,[†,‡] Sameeha Sultana,[†] William Archacki,[¶] Khai-Nghi Truong,[§] Irin E. Aby,[†] Brandon R. Barnett,[†] Luca Catalano,[‖,†] Agnes E. Thorarinsdottir,[*,†] and Michael T. Ruggiero[*,†,⊥,#]

† *Department of Chemistry, University of Rochester, Rochester, NY 14627, USA*

‡*Equal Contribution*

¶ *Department of Chemistry, Yale University, New Haven, CT 06511, USA*

§*Rigaku Europe SE, Hugenottenallee 167, 63263 Neu-Isenburg, Germany*

‖*Dynamic Molecular Materials Laboratory, Department of Life Sciences, University of Modena and Reggio Emilia, Modena 41125, Italy*

⊥*Department of Chemical Engineering, University of Rochester, Rochester, NY 14627, USA*

#*CMAC, Strathclyde Institute of Pharmacy and Biomedical Sciences, University of Strathclyde, Glasgow G4 0RE, UK*

E-mail: Agnes.Thorarinsdottir@rochester.edu; Michael.Ruggiero@rochester.edu

Table of Contents

List of Tables 3

List of Figures 3

Experimental Section 5

Materials and Methods 5

Synthesis 10

Elemental Analysis 12

Powder X-ray Diffraction 12

Fourier Transform Infrared Spectroscopy 12

Nitrogen Adsorption Measurements 12

Electron Diffraction 13

Raman Spectroscopy 15

Terahertz Spectroscopy 15

Calculations 16

CRYSTAL23 16

GAUSSIAN16 16

Supplementary Figures 18

Mode Visualization 36

References 37

List of Tables

S1 Nomenclature for different phases of COF-300 and COF-320. 6
S2 Overview of crystallographic data parameters for COF-300-AR. 7
S3 Overview of crystallographic data quality statistics for COF-300-AR. 7
S4 Crystallographic data and refinement parameters for COF-300-AR. 8
S5 Crystallographic data and refinement parameters for COF-300-H_2O. 9

List of Figures

S1 Synthetic scheme of COF-300-H_2O. 18
S2 Synthetic scheme of COF-300-AR. 18
S3 Synthetic scheme of COF-320A. 18
S4 Comparison of experimental and simulated PXRD patterns of COF-300-H_2O. . . 19
S5 Comparison of experimental and simulated PXRD patterns of COF-320A. 19
S6 FTIR-ATR spectrum of COF-300-H_2O. 20
S7 FTIR-ATR spectrum of COF-320A. 20
S8 N_2 isotherms of COF-300-H_2O at 77 K. 21
S9 Thermogravimetric analysis of COF-300-H_2O. 21
S10 Comparison of Raman spectra of COF-300-H_2O before and after vacuum treatment. 22
S11 N_2 isotherms of COF-320A at 77 K. 22
S12 FTIR-ATR spectrum of COF-300-AR. 23
S13 Comparison of FTIR-ATR spectra between COF-300-H_2O and COF-300-AR. . . 23
S14 Comparison of experimental and simulated PXRD patterns of COF-300-AR. . . . 24
S15 Comparison of PXRD patterns from this work with literature. 24
S16 Crystal structure of open-pore COF-300-AR highlighting packing and linker geometry. 25
S17 Transmission electron microscope images and diffraction patterns of COF-300-AR. 26

S18 Crystal structure of COF-300-AR highlighting thermal ellipsoids of the asymmetric unit. 26
S19 Crystal structure of COF-300-AR. 27
S20 N_2 isotherms of COF-300-AR at 77 K. 28
S21 Variable-temperature Raman spectra of COF-300-H_2O. 28
S22 Variable-temperature Raman spectra of COF-300-AR. 29
S23 Variable-temperature terahertz spectra of COF-300-H_2O. 30
S24 Variable-temperature terahertz spectra of COF-300-AR. 31
S25 Variable-temperature terahertz spectra of COF-320A. 32
S26 Calculated normal-mode displacement patterns for COF-300-H_2O, COF-320A, and COF-300-AR from solid-state DFT vibrational analysis. 33
S27 Comparison of calculated solid-state and gas-phase force constants for COFs. . . 34
S28 Dihedral angle labels for the representative linker portions. 35
S29 Crystal structure of COF-300-AR highlighting C−H$\cdots\pi$ interaction. 35
S30 Rigid potential energy surface scan of the imine (N=C) bond in a representative linker fragment of COF-300 36

Experimental Section

Materials and Methods

General Considerations, Tables, and Nomenclature. Unless otherwise specified, the manipulations described below were carried out under ambient atmosphere and temperature (≈19 °C to 23 °C) using commercial reagents and solvents that were used as received without further purification. COF-320A was obtained by grinding the synthesized powder with mortar and pestle following purification and activation. Samples of COF-300-AR were stored under a dry N_2 atmosphere in a MBraun UNIlab pro glovebox after synthesis. Ultrapure water (18.2 MΩ cm) used for washing COF samples was obtained from an Arium Mini water purification system from Sartorius. Ultrasonic cleaner from DK SONIC (120 W ultrasonic power, 28 kHz to 40 kHz) was used in the synthesis of COF samples. Beckman Coulter Allegra 25R centrifuge was used for purification of COF samples.

Table S1: Nomenclature for different phases of COF-300 and COF-320.

Name	Description
Contracted COF-300-H_2O	Hydrated contracted form of COF-300 studied experimentally in this work. The PXRD pattern is consistent with the hydrated COF-300-H_2O structure, and the sample exhibits negligible N_2 uptake after activation conditions. This phase should therefore be described as a hydrated contracted phase.[1]
COF-300-V	Activated guest-free COF-300 phase reported by Chen *et al.* This phase is included here only for nomenclature and literature comparison, since the experimentally studied COF-300 phase in this work is COF-300-H_2O.[1]
Collapsed COF-300-AR	Reduced COF-300 derivative featuring amine bonds studied experimentally in this work. The solved structure corresponds to a collapsed packing arrangement and the N_2 isotherm shows negligible gas uptake.
Open-pore COF-300-AR	Previously reported open-pore reduced COF-300 structure included here for structural comparison. This phase has a more linear linker geometry and is not the experimentally studied COF-300-AR phase in this work.[2]
Contracted, guest-responsive COF-320A	Extended atropisomeric COF-320 analogue studied experimentally in this work. The starting structure corresponds to a contracted phase, while the N_2 isotherm shows a stepped gas uptake consistent with guest-responsive pore opening. Gas-triggered structural flexibility of COF-320A was reported by Kang *et al.*[3]

Table S2: Overview of crystallographic data collection parameters for COF-300-AR.

Dataset	Scan range (°)	Scan width (°)	Number of frames	Exposure time per frame (s)	Total exposure time (s)	Dose rate (e^- ($Å^2$ s)$^{-1}$)	Dose (e^- $Å^{-2}$)
**10316**	−62 to +63	0.50	250	0.50	125	5.18×10^{-3}	0.65
**10318**	−60 to +60	0.50	240	0.50	120	50.24×10^{-3}	0.63
**10319**	−63 to +65	0.50	256	0.50	128	5.23×10^{-3}	0.67

Table S3: Overview of crystallographic data quality statistics for all single and merged data collections for COF-300-AR. All datasets were processed up to a resolution of 0.80 Å. Point group symmetry: *Fdd*2.

Dataset (grain)	Data	Compl. %	Redund.	$\langle F2 \rangle$	$\langle F2/\sigma(F2) \rangle$	R_{int}	R_{rim}	R_{pim}	$CC_{1/2}$
**10316**	7999	93.7	2.7	4109.66	3.43	0.192	0.135	0.245	0.964
**10318**	7633	95.2	4.6	7823.82	7.61	0.120	0.064	0.145	0.993
**10319**	7849	93.8	2.6	6684.96	3.79	0.186	0.132	0.254	0.946
**merged**	22894	97.3	7.4	5726.06	7.05	0.187	0.073	0.217	0.987

Table S4: Crystallographic data and refinement parameters for COF-300-AR, CCDC 2516800, $C_{164}H_{144}N_{16}$.

Parameter	Value
Formula weight, M_r	2339.07 g mol^{-1}
Crystal system	Orthorhombic
Space group	*Fdd2* (No. 43)
a	40.748(2) Å
b	20.830(3) Å
c	7.0492(4) Å
α	90°
β	90°
γ	90°
V	5983.3(10) Å^3
Z	2
Z'	0.125
T	175 K
μ (TEM)	0.000
Total reflections	22314
Observed reflections, $I_0 > 2\sigma(I_0)$	2220
Resolution	0.80 Å
Completeness	96.2%
R_{int}	0.1874
Data	2980
Parameters	205
Restraints	322
GOF	1.627
R_1, wR_2 [$I_0 > 2\sigma(I_0)$]	0.1270, 0.2959
R_1, wR_2 [all reflections]	0.1550, 0.3063
Residual density, $\Delta\rho_{min}/\Delta\rho_{max}$	$-0.174/0.178$

Table S5: Crystallographic data and refinement parameters for COF-300-H_2O.

Parameter	Value
Formula weight, M_r	1297.53 g mol^{-1}
Crystal system	Tetragonal
Space group	$I4_1/a$ (No. 88)
a	19.4641(15) Å
b	19.4641(15) Å
c	8.9595(9) Å
α	90°
β	90°
γ	90°
V	3394.3(6) Å^3
Z	2
T	175 K
Total measured reflections	8825
Unique reflections	1725
Observed reflections, $I > 2\sigma(I)$	1514
R_{int}	0.1726
Parameters	120
Restraints	153
GOF	1.180
R_1 [$I > 2\sigma(I)$]	0.2059
R_1 [all reflections]	0.2188
wR_2 [all reflections]	0.4506
Residual density, $\Delta\rho_{\text{min}}/\Delta\rho_{\text{max}}$	$-0.199/0.161$

Synthesis

Synthesis of COF-300-H_2O. The material was synthesized following a modified literature procedure.[4] A 50 mL Falcon conical centrifuge tube was charged with tetrakis(4-aminophenyl)methane (40.9 mg, 0.11 mmol), terephthalaldehyde (24.2 mg, 0.18 mmol), and 1,4-dioxane (2 mL) to form a white suspension. The suspension was sonicated for 10 minutes (28 kHz, 25 °C), until all solids had dissolved. To the solution, aniline (0.12 mL, 1.32 mmol) was added, followed by glacial acetic acid (0.40 mL, 6.99 mmol). The resulting mixture was left undisturbed in sealed tubes at ambient temperature for 72 hours to give a yellow precipitate. The solid was collected by centrifugation (5 minutes, 9000 rpm) and washed with 1,4-dioxane (15 mL) and ethanol (2 x 15 mL) after discarding the supernatant. For each wash, the solid was mixed with solvent and the supernatant was discarded after isolating the solid by centrifugation (5 minutes, 9000 rpm). The resulting yellow powder was dried at 65 °C for 16 hours to give the hydrated contracted phase of COF-300, COF-300-H_2O (49.7 mg, 89 %). Anal. Calcd. for $C_{41}H_{28}N_4 \cdot 2.5H_2O$: C, 79.20; H, 5.35; N, 9.01 %. Found: C, 79.25; H, 5.24; N, 8.94 %. FTIR (ATR, cm^{-1}): 3377 (w), 3263 (w), 3064 (w), 3032 (w), 2903 (w), 1661 (w), 1616 (m), 1586 (m), 1575 (w), 1496 (m), 1417 (w), 1377 (w), 1363 (w), 1321 (w), 1305 (w), 1286 (w), 1225 (w), 1199 (w), 1176 (m), 1127 (w), 1113 (w), 1015 (w).

Synthesis of COF-300-AR. The material was synthesized following a modified literature procedure.[5] A 50 mL round bottom flask was charged with a Teflon-lined stir bar, COF-300-H_2O (51.4 mg, 0.083 mmol) and methanol (25 mL) to form a yellow suspension. To the resulting suspension, *p*-phthalic acid (58.0 mg, 0.35 mmol) was added and the mixture was placed in an ice - water bath and stirred for 5 minutes. To the mixture, sodium borohydride (507.5 mg, 13.4 mmol) was added as a solid in small portions over the course of 10 minutes. After the addition was completed, the resulting white suspension was stirred in the ice-water bath for 1 hour, followed by stirring at ambient temperature for additional 16 hours. The white solid was collected by centrifugation (5 minutes, 9000 rpm) and washed with ultrapure water (3 x 15 mL to 20 mL) after

discarding the supernatant. For each wash, the solid was mixed with ultrapure water and the supernatant was discarded after isolating the solid by centrifugation (5 minutes, 9000 rpm). The white solid was dried at 65 °C for 16 hours to give COF-300-AR (36.2 mg, 72 %). Anal. Calcd. for $C_{41}H_{36}N_4 \cdot 1.5H_2O$: C, 80.49; H, 6.43; N, 9.16 %. Found: C, 80.05; H, 6.06; N, 9.02 %. FTIR (ATR, cm^{-1}): 3425 (w), 3365 (w), 3048 (w), 3022 (w), 2852 (w), 1608 (m), 1579 (w), 1511 (s), 1461 (m), 1417 (w), 1403 (w), 1357 (w), 1332 (w), 1319 (w), 1299 (w), 1276 (w), 1258 (m), 1185 (m), 1128 (w), 1115 (w), 1019 (w).

Synthesis of COF-320A. The material was synthesized following a modified literature procedure.[6] A 20 mL scintillation vial was charged with tetrakis(4-aminophenyl)methane (46 mg, 0.05 mmol), 4,4'-biphenyldialdehyde (42.0 mg, 0.10 mmol), 1,4-dioxane (0.5 mL), and pyridine (0.3 mL) to form a yellow suspension. The suspension was sonicated for 10 minutes (28 kHz, 25 °C), until all solids had dissolved. To the solution, aniline (0.12 mL, 1.32 mmol) was added, followed by 6 M acetic acid solution in water (0.40 mL, 2.40 mmol). The resulting mixture was heated at 85 °C for 72 hours to give a white precipitate. The solid was collected by centrifugation (5 minutes, 9000 rpm) and washed with 1,4-dioxane (3 x 20 mL) and THF (3 x 20 mL) after discarding the supernatant. For each wash, the solid was mixed with solvent and the supernatant was discarded after isolating the solid by centrifugation (5 minutes, 9000 rpm). The white powder was dried at 120 °C under vacuum for 24 hours and acquired a yellow color. After activation, the yellow powder was ground with a mortar and pestle to obtain the contracted phase of the flexible atropisomer of COF-320, COF-320A (60.2 mg, 83 %). Anal. Calcd. for $C_{53}H_{36}N_4 \cdot 3H_2O$: C, 81.31; H, 5.41; N, 7.16 %. Found: C, 81.40; H, 5.09; N, 7.18 %. FTIR (ATR, cm^{-1}): 3027 (w), 2872 (w), 1914 (w), 1620 (m), 1595 (m), 1563 (m), 1496 (m), 1417 (w), 1402 (w), 1354 (w), 1306 (w), 1278 (w), 1161 (m), 1107 (w), 1000 (m), 972 (w), 949 (w), 886 (m), 804 (s), 725 (m).

Elemental Analysis

Elemental analyses of frameworks were performed at the CENTC Elemental Analysis Facility at the University of Rochester. Samples for analysis were heated at 120 °C under dynamic vacuum for 16 hours prior to being weighed with a PerkinElmer Model AD 6000 Autobalance. Elemental compositions were determined with a PerkinElmer 2400 Series II Elemental Analyzer.

Powder X-ray Diffraction

Powder X-ray diffraction (PXRD) was performed on a Rigaku MiniFlex 6G diffractometer (Cu Kα, λ = 1.5418 Å, 40 kV, 15 mA) in Bragg–Brentano geometry. Patterns were recorded over 2θ = 5° to 30° with a 0.01° step size and a 1° per minute scan rate. Diffractograms were processed with SmartLab Studio II. An asymmetric least squares baseline correction was applied to account for air scattering at low 2θ angles.

Fourier Transform Infrared Spectroscopy

Fourier Transform Infrared (FTIR) spectra of COF samples were collected using a PerkinElmer Spectrum 3 FTIR spectrometer. Dried solid samples were placed onto a Universal diamond attenuated total reflectance (ATR) top-plate and measured using 16 scans in the 550 cm^{-1} to 4000 cm^{-1} range with 1 cm^{-1} resolution. Background correction was applied using ambient air as the background, measured using 16 scans.

Nitrogen Adsorption Measurements

Nitrogen adsorption isotherm measurements were performed on a Micromeritics 3Flex physisorption analyzer using ultrahigh purity N_2 gas (99.99%) at 77 K. An oven-dried analysis tube with a glass filler rod was evacuated on the adsorption analyzer for 12 hours, refilled with N_2 gas (1000 mbar), and the mass of the tube and rod was obtained. Samples for analysis were activated by heating at 120 °C under dynamic vacuum for 12 hours, followed by activation at 120 °C under dynamic vacuum in the analysis tube on the adsorption analyzer for 12 hours. After activation,

the tube was refilled with N_2 gas (1000 mbar), and the exact mass of the activated sample was obtained. The analysis tube, equipped with the rod and sample, was then attached to the analysis port of the physisorption analyzer for 77 K N_2 surface area measurement. A liquid N_2 cooling bath was used to keep the measurement temperature at 77 K. The surface area measurements indicated negligible adsorption of N_2 for COF-300-H_2O and COF-300-AR.

Single Crystal Structure Determination via 3D Electron Diffraction

Electrons feature very strong interactions with the electrostatic potential of atoms. Subsequently, electron diffraction allows for performing experiments with crystallites in the nanometer range. However, it needs to be considered that the absorption of the samples is much stronger than in X-ray diffraction and the data are affected by dynamical diffraction as well as ionic scattering factors. This can lead to seemingly bad R-values for the refinement in the simplistic kinematic approximation.

Microcrystalline COF-300-H_2O was examined by continuous-rotation 3D electron diffraction at 175 K using electron radiation ($\lambda = 0.0251$ Å) and a HyPix-ED detector. The dataset was collected over a rotation range of $-63°$ to $+64°$ using a 0.50° frame width and 0.50 s exposure time per frame, yielding 254 frames. The diffraction data were processed using CrysAlisPro, and the structure was solved using ShelXT and refined in the kinematical approximation using ShelXL within Olex2. The structure was refined in the tetragonal $I4_1/a$ space group with unit-cell parameters $a = b = 19.4641(15)$ Å, $c = 8.9595(9)$ Å, and $V = 3394.3(6)$ Å^3. The comparatively high refinement residuals arise primarily from dynamical scattering in the electron-diffraction data from the relatively thick crystallite (>500 nm), while the structure was refined using the kinematical approximation. The resulting structure is consistent with the hydrated contracted COF-300 phase previously reported in literature[1,7–13] and a CSD reduced-cell search identified XOCXEV as a closely matching entry[1]. The crystallographic data and refinement parameters are summarized in **Table S5**.

For COF-300-AR, microcrystalline powder was spread on a lacey-carbon-supported copper

TEM grid. Yellowish plate-like crystallites with a few 100 nm thickness were selected for 3D-ED/microED measurements. Cryo-transfer, i.e. freezing of samples prior to introduction to vacuum, at 175 K using a Gatan ELSA (Model 698) specimen holder was applied here. As electron diffraction requires samples to be studied under ultra-high vacuum, the cryo-transfer technique is essential for many sensitive compounds, such as solvent-containing MOFs, COFs or proteins. In addition to stabilization in vacuo, other benefits are improving resolution, reducing disorder, and reducing beam damage. Different from the hydrated contracted COF-300-H_2O framework, COF-300-AR crystallizes in its anhydrous form.

Electron diffraction measurements for COF-300-AR were collected using the Rigaku XtaLAB Synergy-ED, equipped with a Rigaku HyPix-ED detector optimized for operation in the continuous rotation 3D-ED experimental setup.[14,15] Data acquisition was performed at 175 K under high vacuum with an electron wavelength of 0.0251 Å (200 kV). The instrument was operated and the diffraction data were processed in the program CrysAlisPro.[16] A multi-scan absorption correction was performed using spherical harmonics implemented in the SCALE3 ABSPACK scaling algorithm in CrysAlisPro. The structure was solved using ShelXT,[17] and subsequently, refined with kinematical approximation using ShelXL[18] in the crystallographic program suite Olex2.[19,20] By merging data of three individual grains/datasets, a completeness of 96.2% up to a resolution of 0.80 Å was achieved. Non-hydrogen atoms were assigned anisotropic displacement parameters. The hydrogen atoms bonded to nitrogen atoms were located from Fourier difference maps. Other hydrogen atoms were placed in idealized positions and included as a riding model. Isotropic displacement parameters for all H atoms were constrained to multiples of the equivalent displacement parameters of their parent atoms with Uiso(H) = 1.2 Ueq(parent atom). A few reflections with large discrepancies between the calculated and observed structure factors have been omitted from the least-squares refinement as outliers. Enhanced rigid bond restraints[21,22] with standard uncertainties of 0.001 $Å^2$ were applied. The experimental and refinement details are given below. CCDC 2516800 contains the supplementary crystallographic data for this publication. These data can be obtained free of charge via www.ccdc.cam.ac.uk/data_request/cif, or by emailing

data_request@ccdc.cam.ac.uk, or by contacting The Cambridge Crystallographic Data Centre, 12 Union Road, Cambridge CB2 1EZ, UK; fax: +44 1223 336033.

Raman Spectroscopy

Raman spectra were collected on a THz-Raman system (Coherent Ondax; 785 nm excitation wavelength) coupled to an Andor Shamrock SR-500i spectrograph and iDus 416 CCD detector. Finely ground microcrystalline samples were measured after activation under vacuum at 373 K for 16 hours in a continuous-flow cryostat (Lake Shore Cryotronics) under vacuum (1×10^{-3} mbar). Spectra were acquired every 10 K from 80 K to 300 K; each spectrum was averaged over 10 scans with 10 s integration time. The measured Stokes intensities were corrected for the Bose–Einstein thermal population factor, $n(\omega, T) + 1$, to account for the temperature dependence of phonon population.

Terahertz Spectroscopy

Samples for terahertz spectroscopy measurements were finely ground and mixed with PTFE powder (Howard Piano Industries) in a 2 wt.% ratio and pressed into 3 mm free standing pellets. Blanks were prepared with the same amount of PTFE and also pressed into pellets. Terahertz spectra were acquired after activation under vacuum at 373 K for 16 hours in a continuous-flow cryostat (Lake Shore Cryotronics) under vacuum (1×10^{-3} mbar) using a Teraflash Pro (Toptica) in transmission geometry while purging with nitrogen gas. The temperature was controlled by a cryostat (Lake Shore Cryotronics), and samples were allowed to equilibrate for 15 minutes at each temperature prior to measurements. 5000 scans were averaged for samples and references at each temperature (80 K to 300 K), and absorbance was calculated from the Fourier transformed waveforms.[23]

Calculations

CRYSTAL23

Geometry optimizations and vibrational mode assignments were performed using fully periodic solid-state density functional theory with the CRYSTAL23 software package.[24] All calculations were performed at the Ahlrichs-pVTZ/PBE level of theory, including Grimme's D3 dispersion correction.[25–28] Structures obtained from electron diffraction experiments were used as a starting point for all calculations. The structures first underwent a fully unconstrained geometry optimization, with an energy convergence criterion of $\Delta E < 10^{-8}$ Hartree.[29] Subsequently, vibrational frequencies were calculated numerically at the Γ-point, within the harmonic approximation, with an energy convergence criterion of $\Delta E < 10^{-11}$ Hartree.[30,31] The Berry phase and Coupled-Perturbed Hartree–Fock/Kohn–Sham methods were used to calculate IR and Raman intensities, respectively.[32,33] 1D potential energy surfaces were created by scanning the energy along the coordinate of each vibration in steps of 0.25 displacements, where the energy of 1 displacement is equal to the zero-point energy. A normal mode analysis was calculated using the keyword LOCMODES.[34,35]

GAUSSIAN16

Potential energy scans around the N−C and N=C bonds were performed with GAUSSIAN16.[36] All calculations were carried out at the same level of theory as the CRYSTAL23 calculations (Ahlrichs-pVTZ/PBE-GD3BJ), with an energy convergence of $\Delta E < 10^{-11}$ Hartree. The linker geometry was simulated by extracting a single linker portion from the ED structure, and replacing the node carbons with a hydrogen atom. The positions of hydrogen atoms were then optimized. In the rigid scan, structures were manually generated by sequentially varying the φ torsion angles with Rodrigues' rotation formula. For the relaxed structures the Opt=ModRedundant keyword was used, where the φ angles were frozen, while the rest of the geometry was allowed to fully relax.

The gas phase local mode analysis was performed following the approach of Konkoli and Cremer.[37,38] For each linker, a full geometry optimization was carried out followed by a frequency calculation at the same level of theory as before (Ahlrichs-pVTZ/PBE-GD3BJ).

The Hessian matrix and equilibrium geometry were extracted from the GAUSSIAN16 formatted checkpoint file. For a given internal coordinate q_n, the associated local vibrational mode $\mathbf{a}_n$ was obtained from

$$\mathbf{a}_n = \frac{\mathbf{K}^{-1}\mathbf{d}_n^{\mathsf{T}}}{\mathbf{d}_n\,\mathbf{K}^{-1}\,\mathbf{d}_n^{\mathsf{T}}} \tag{1}$$

where $\mathbf{d}_n = \mathbf{B}_n\mathbf{L}$ is the row of the normal-mode matrix expressed in the internal coordinate q_n, and $\mathbf{B}_n$ is the corresponding row of the Wilson $\mathbf{B}$-matrix. The local mode force constant k_n^a was then evaluated as

$$k_n^a = \left(\mathbf{d}_n\,\mathbf{K}^{-1}\,\mathbf{d}_n^{\mathsf{T}}\right)^{-1} = \left(\sum_{\mu} \frac{D_{n\mu}^2}{k_\mu}\right)^{-1} \tag{2}$$

The local mode analysis was carried out using an in-house Python code, and validated against the analytical diatomic limit, in which the local stretching force constant reproduces the harmonic force constant exactly.

Supplementary Figures

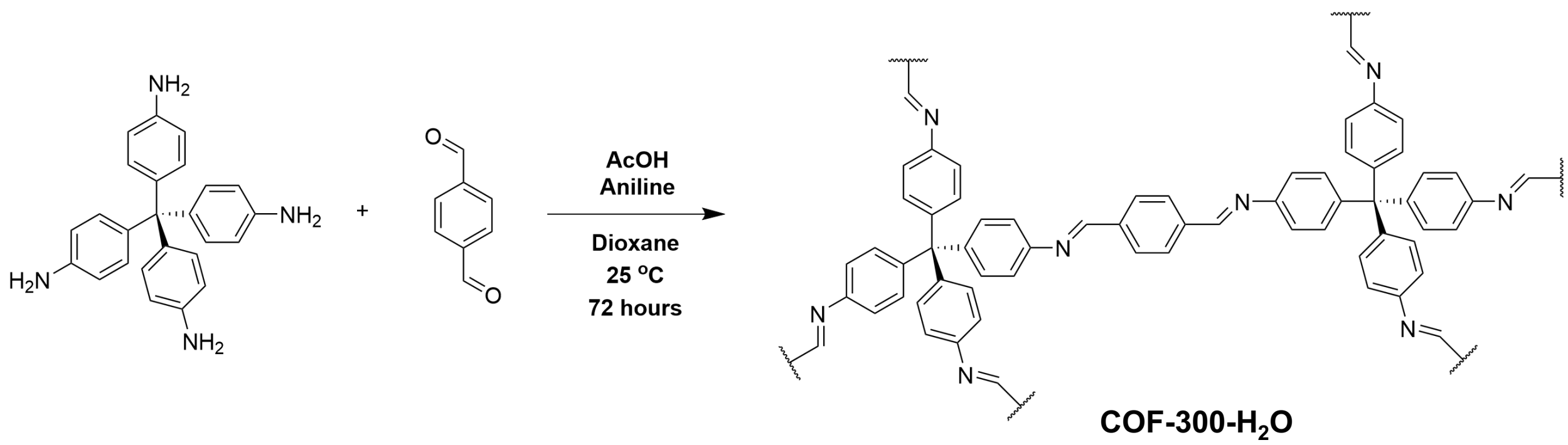

Figure S1: Synthetic scheme of COF-300-H_2O.

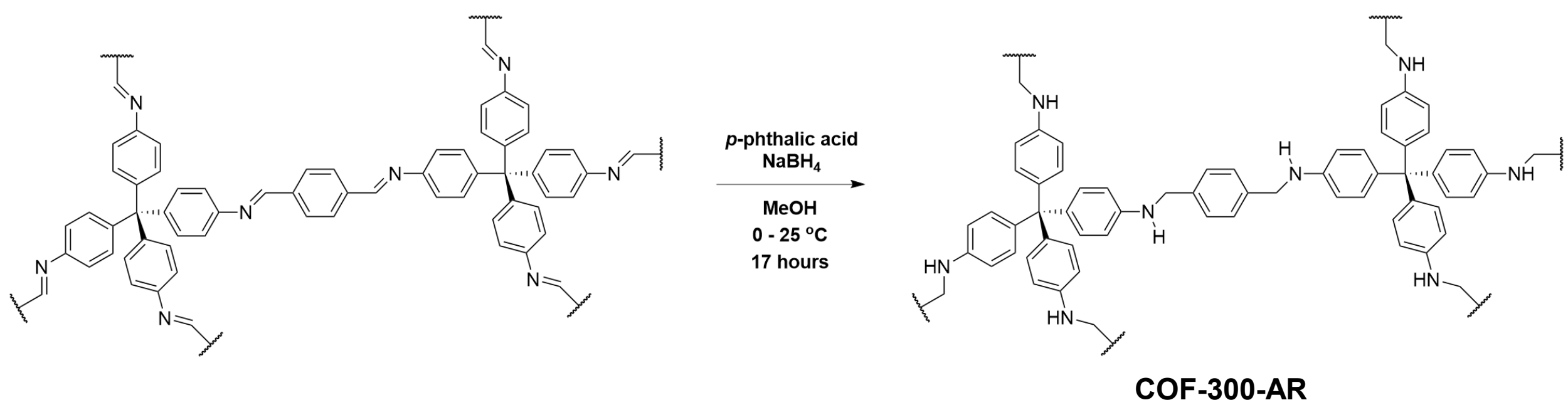

Figure S2: Synthetic scheme of COF-300-AR obtained from post-synthetic modification of COF-300-H_2O.

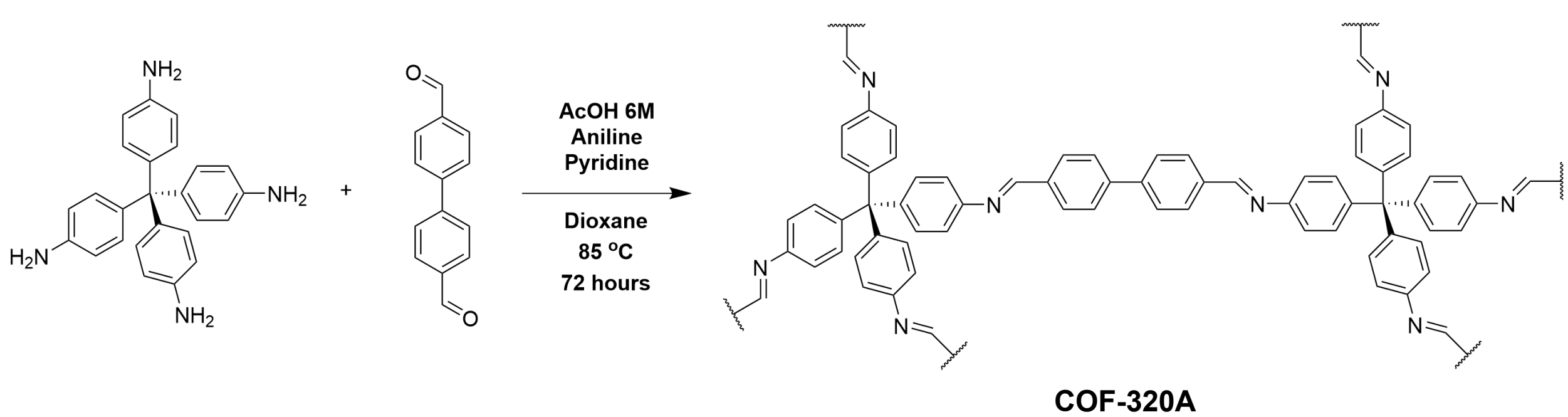

Figure S3: Synthetic scheme of COF-320A.

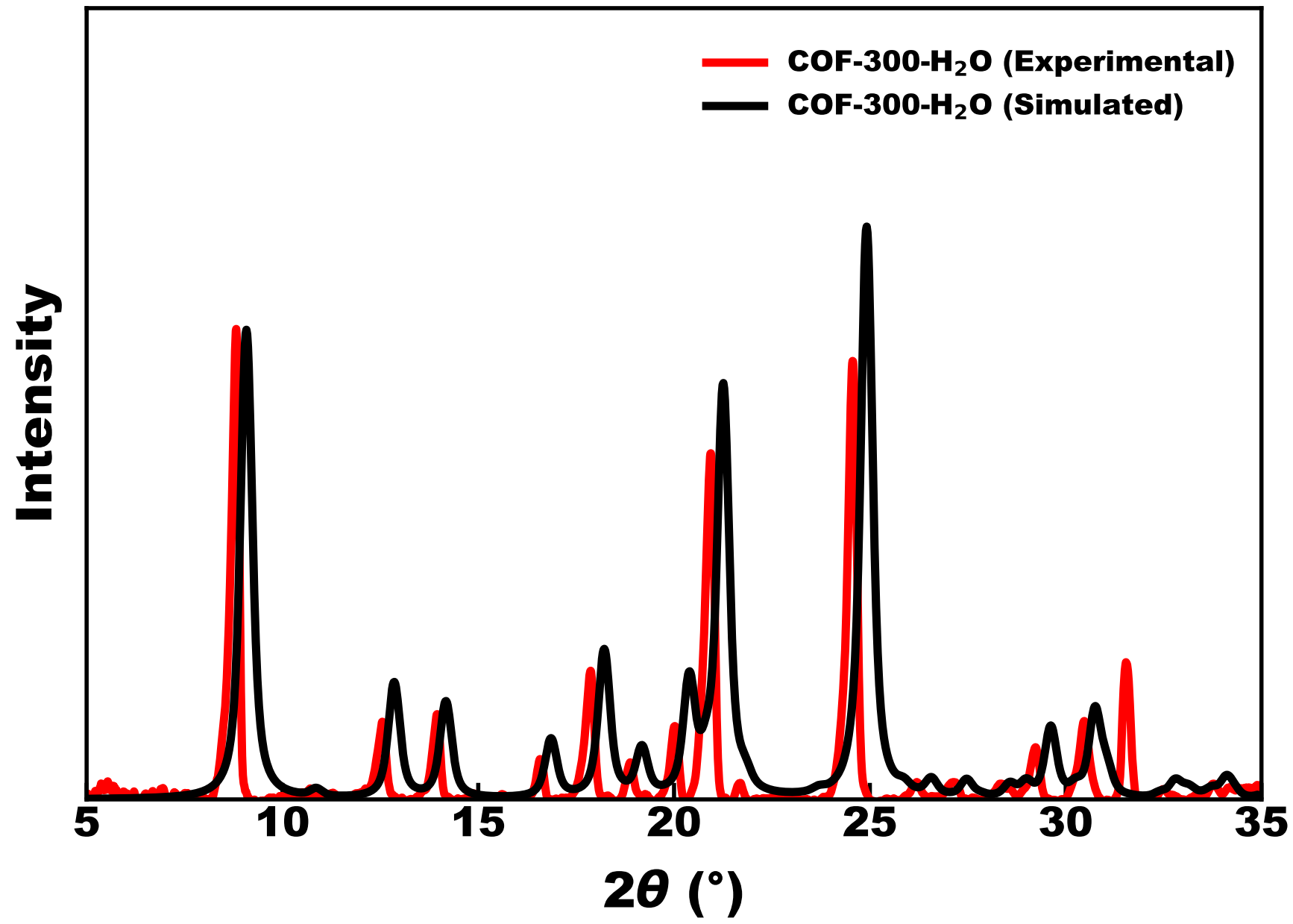

Figure S4: Comparison of experimental (red) and simulated (black) PXRD patterns of COF-300-H_2O.

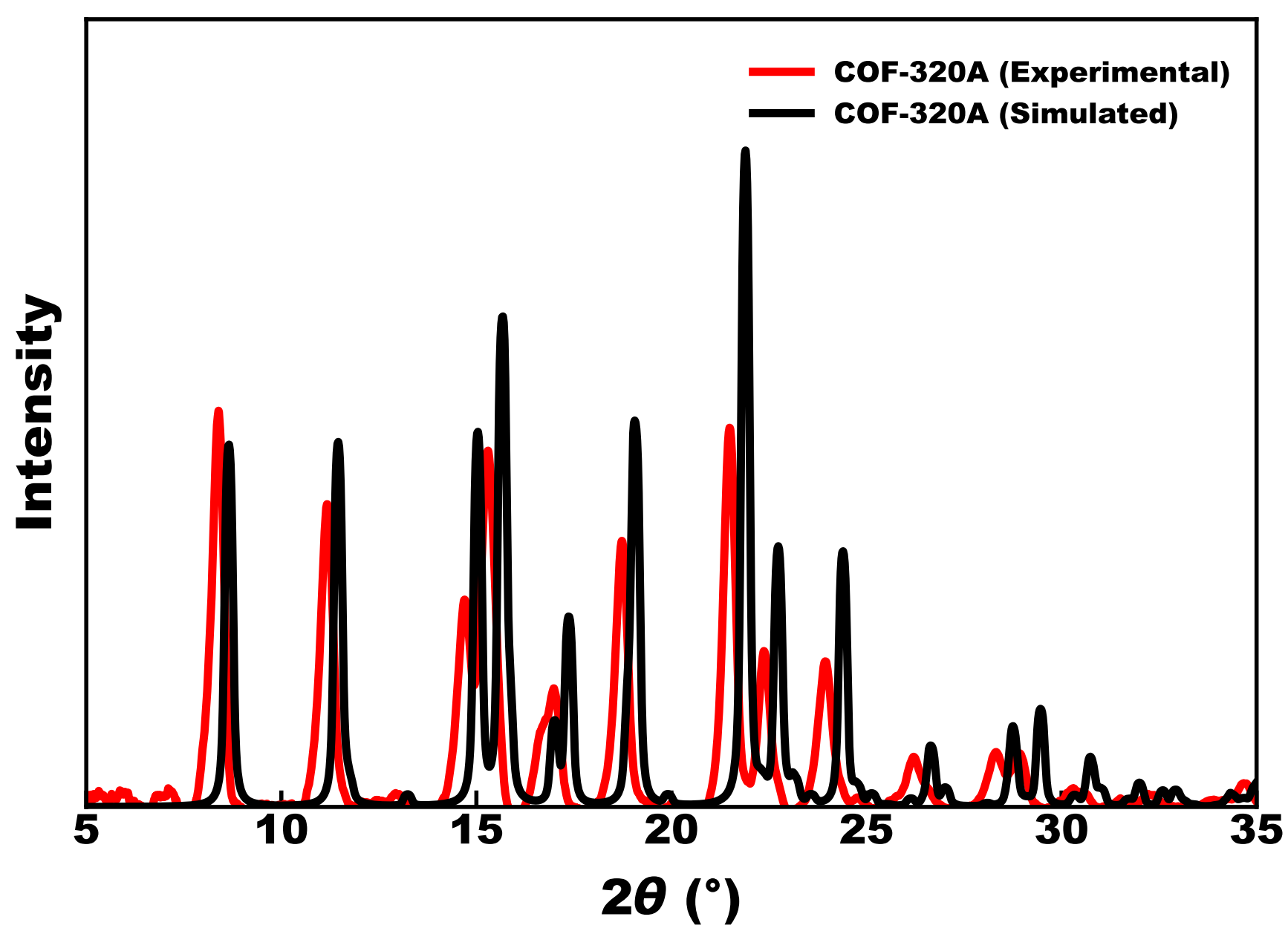

Figure S5: Comparison of experimental (red) and simulated (black) PXRD patterns of COF-320A.

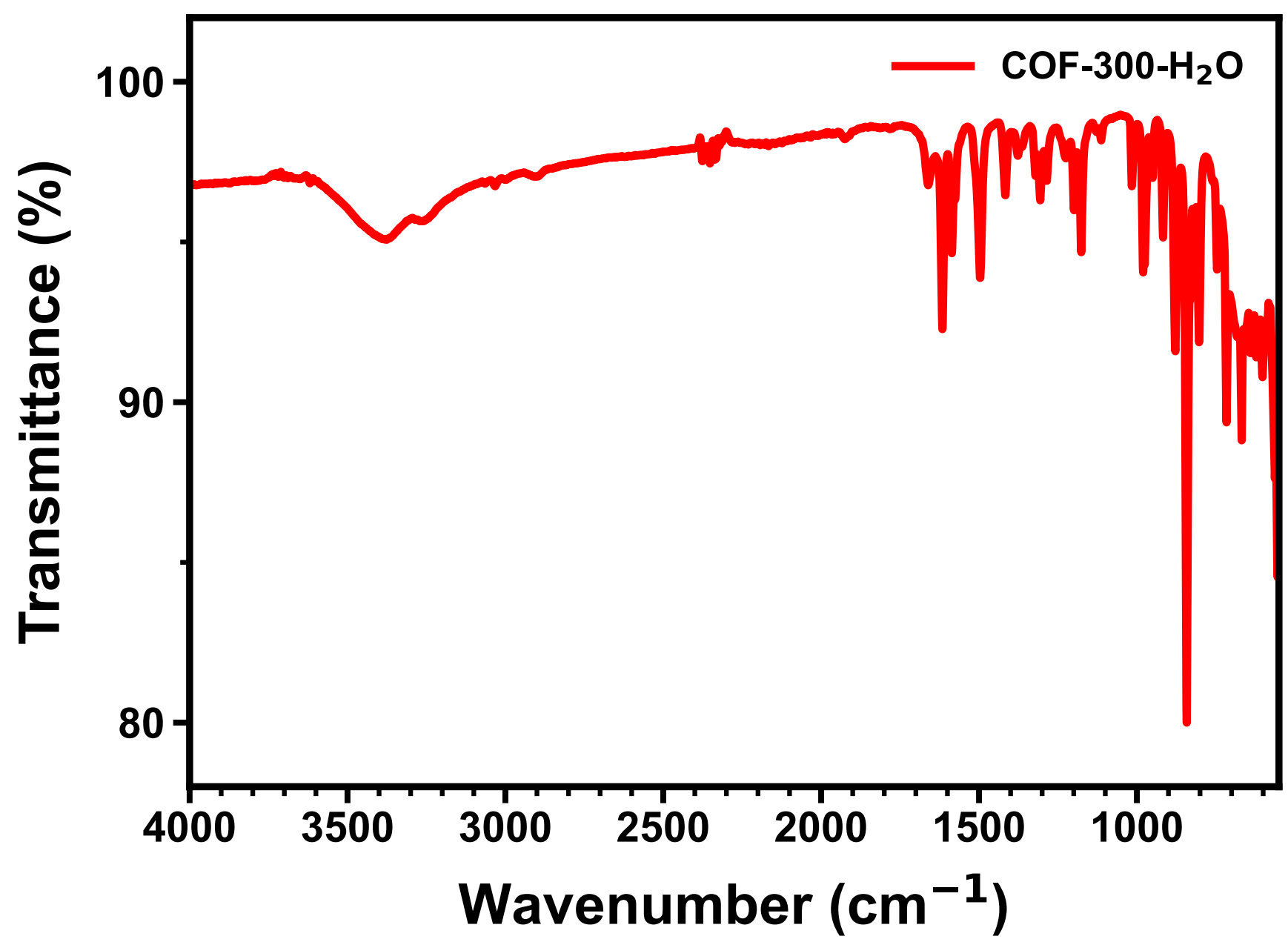

Figure S6: FTIR-ATR spectrum of COF-300-H_2O.

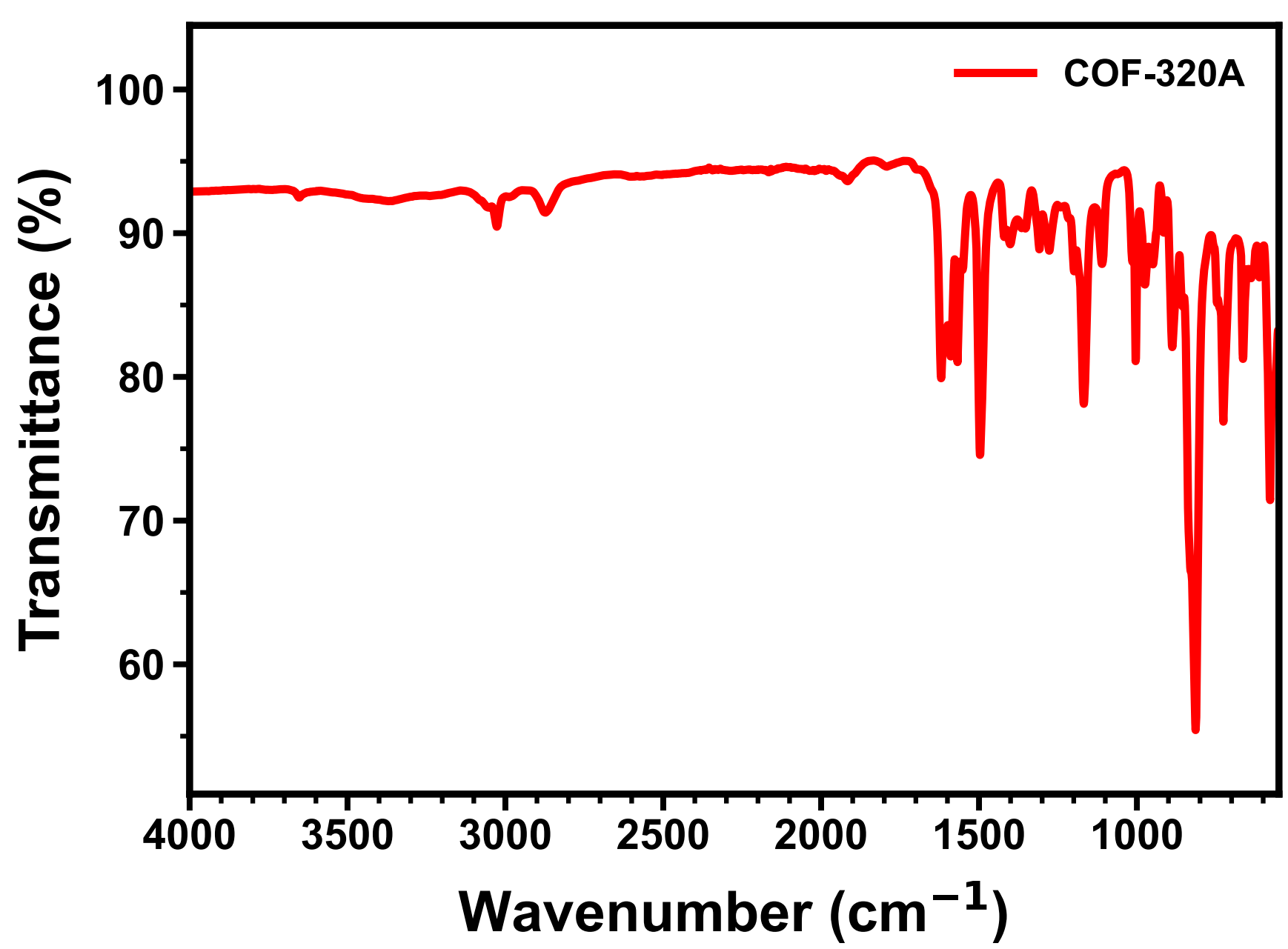

Figure S7: FTIR-ATR spectrum of COF-320A.

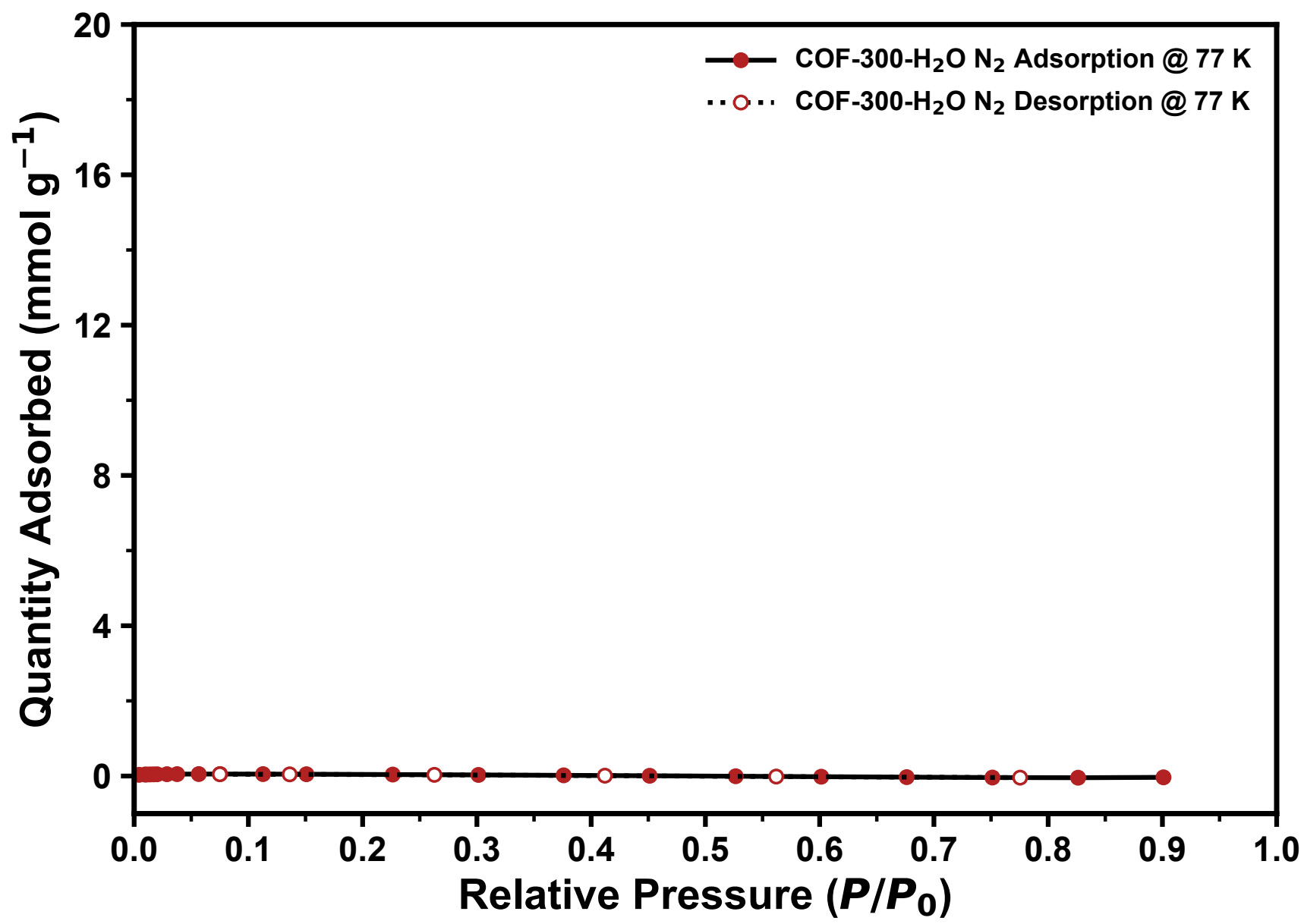

Figure S8: N_2 isotherms of COF-300-H_2O at 77 K. The closed red data points correspond to adsorption points and the open red points correspond to desorption points.

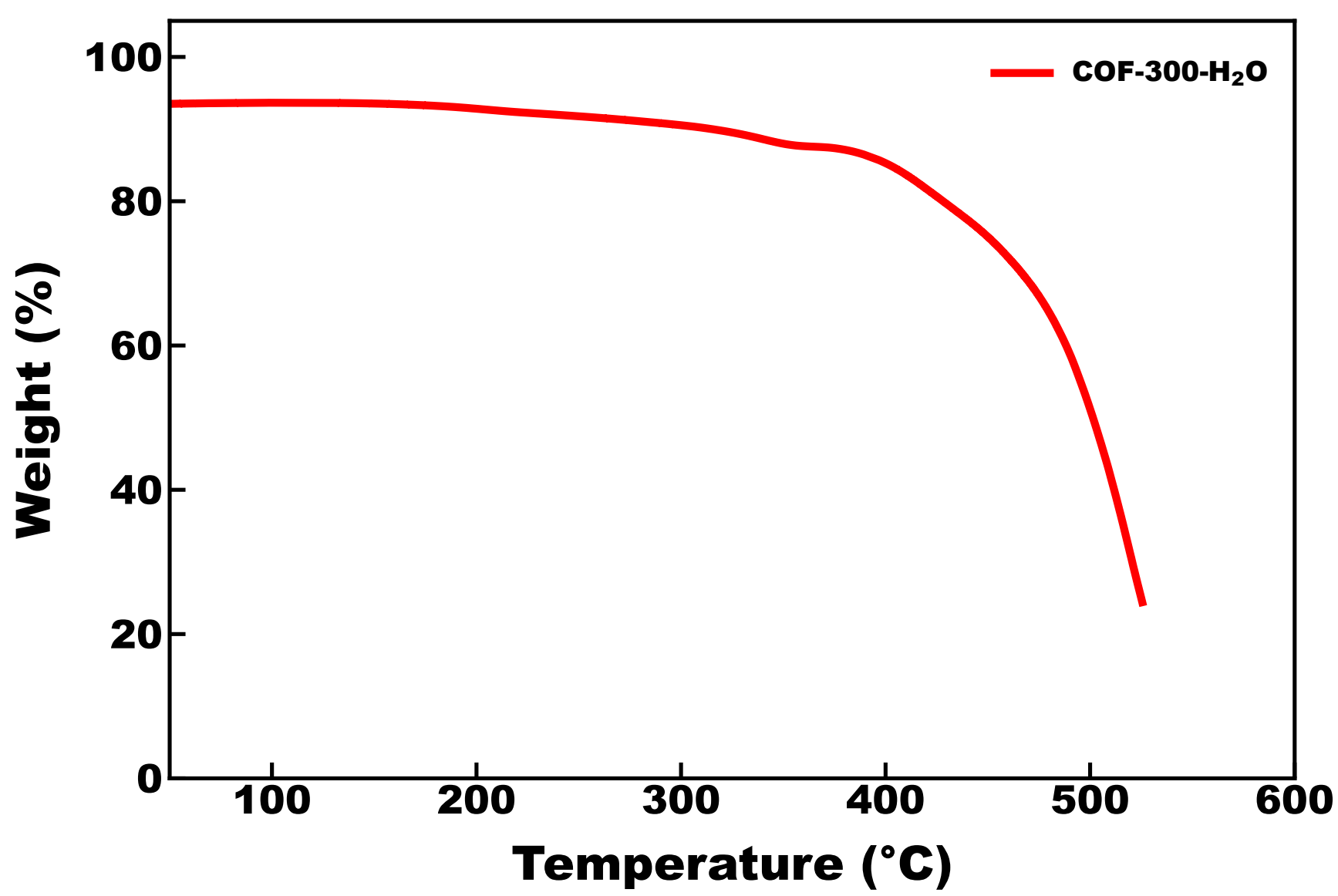

Figure S9: Thermogravimetric analysis of COF-300-H_2O. The sample was equilibrated at 50 °C for 30 min under N_2 atmosphere, followed by heating from 50 °C at 1 °C min^{-1}. No distinct additional low-temperature mass-loss event is observed through the temperature range relevant to the activation conditions used in this work.

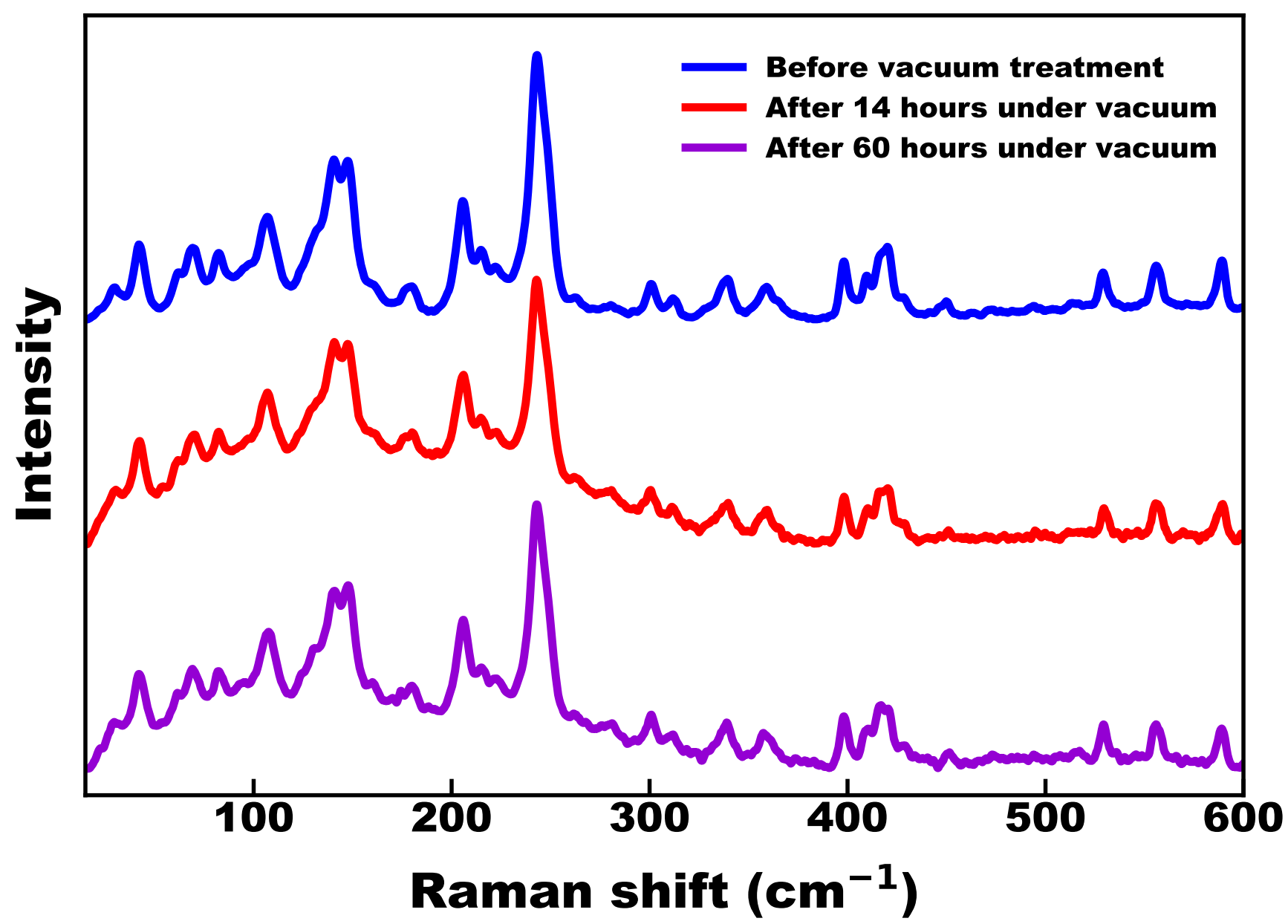

Figure S10: Comparison of Raman spectra of COF-300-H_2O before and after vacuum treatment in the cryostat used for the measurements.

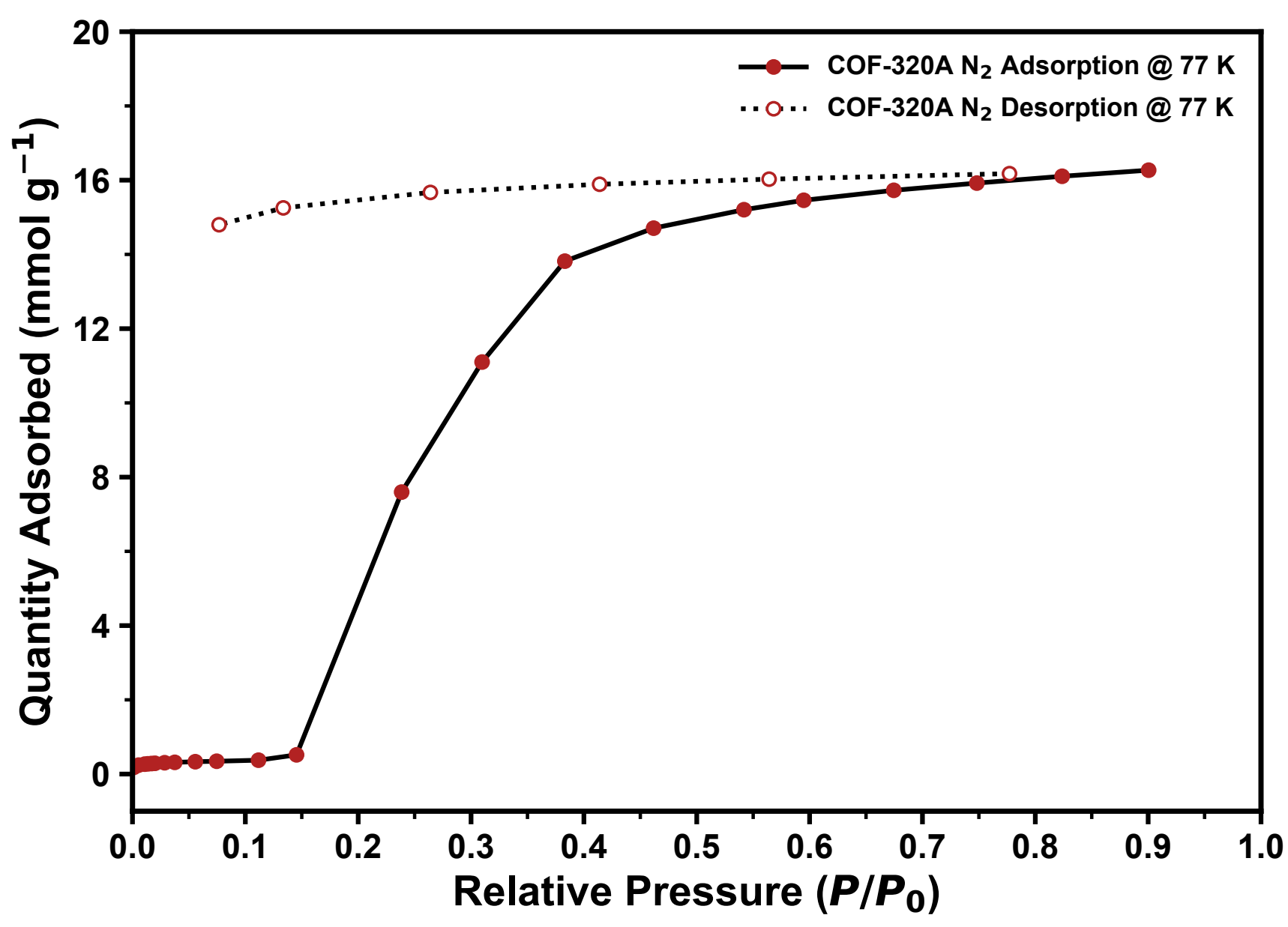

Figure S11: N_2 isotherms of COF-320A at 77 K. The closed red data points correspond to adsorption points and the open red points correspond to desorption points.

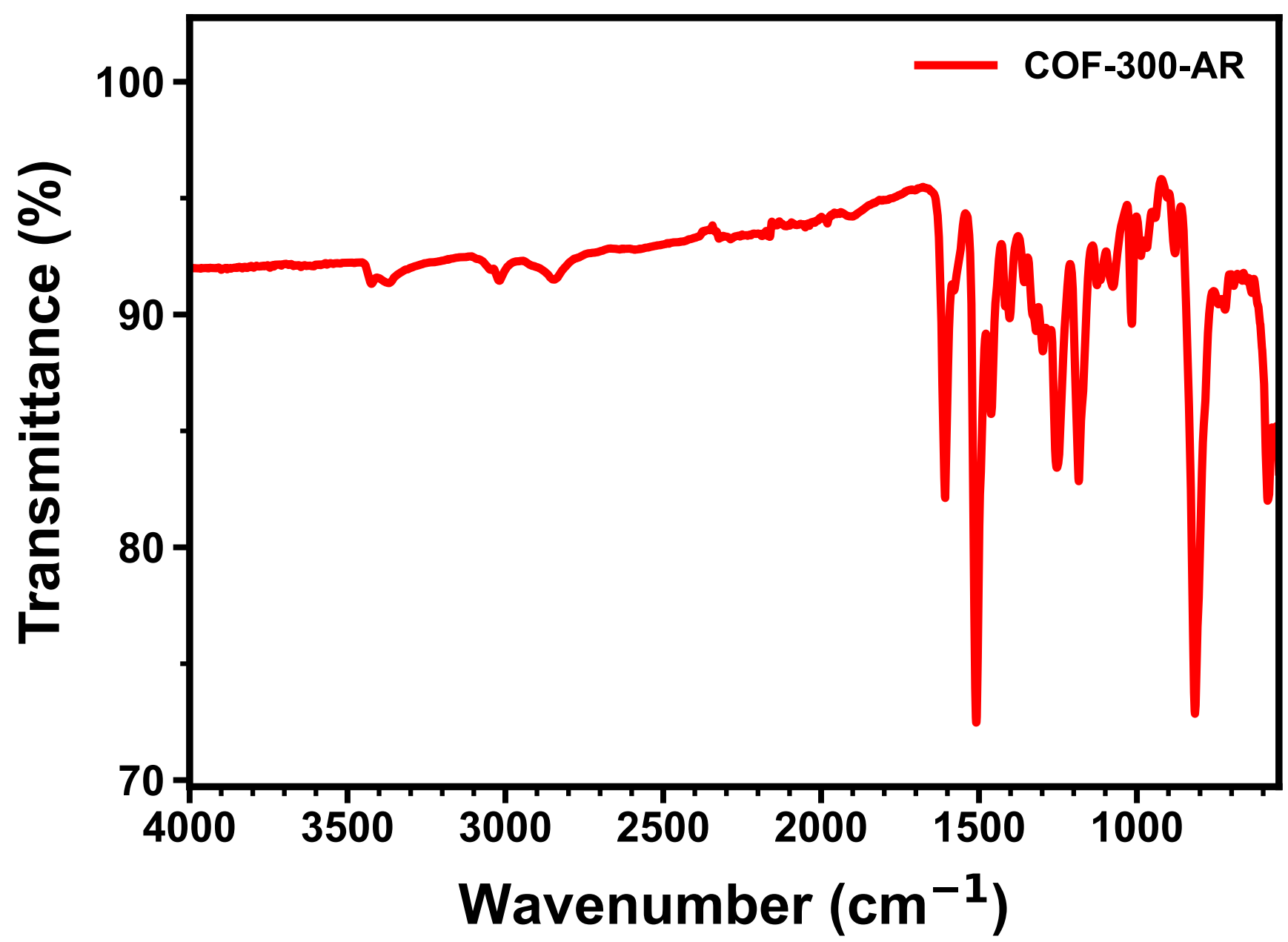

Figure S12: FTIR-ATR spectrum of COF-300-AR.

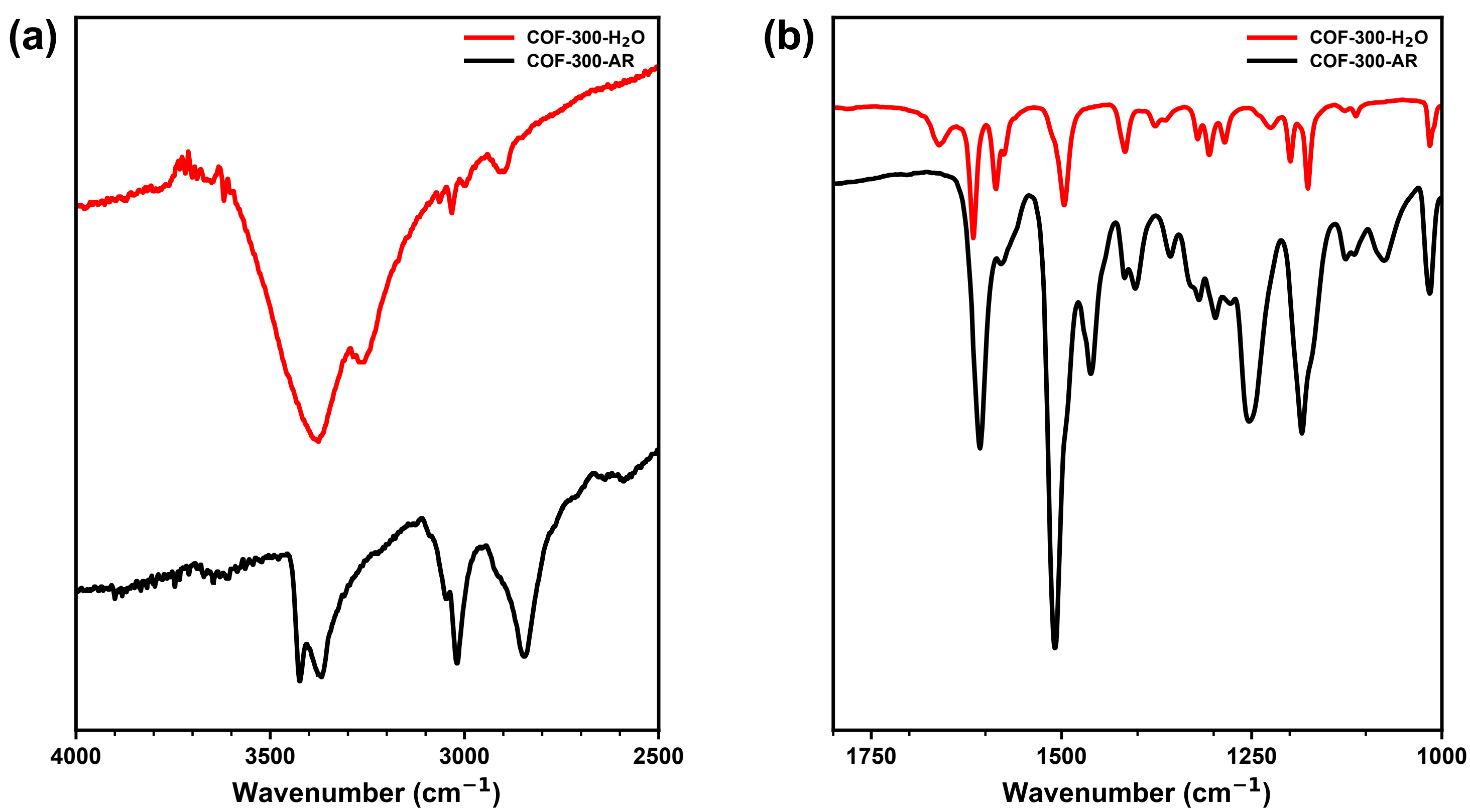

Figure S13: Comparison of FTIR-ATR spectra between COF-300-H_2O (top, red) and COF-300-AR (bottom, black) in the high-energy (a) and low-energy (b) regions.

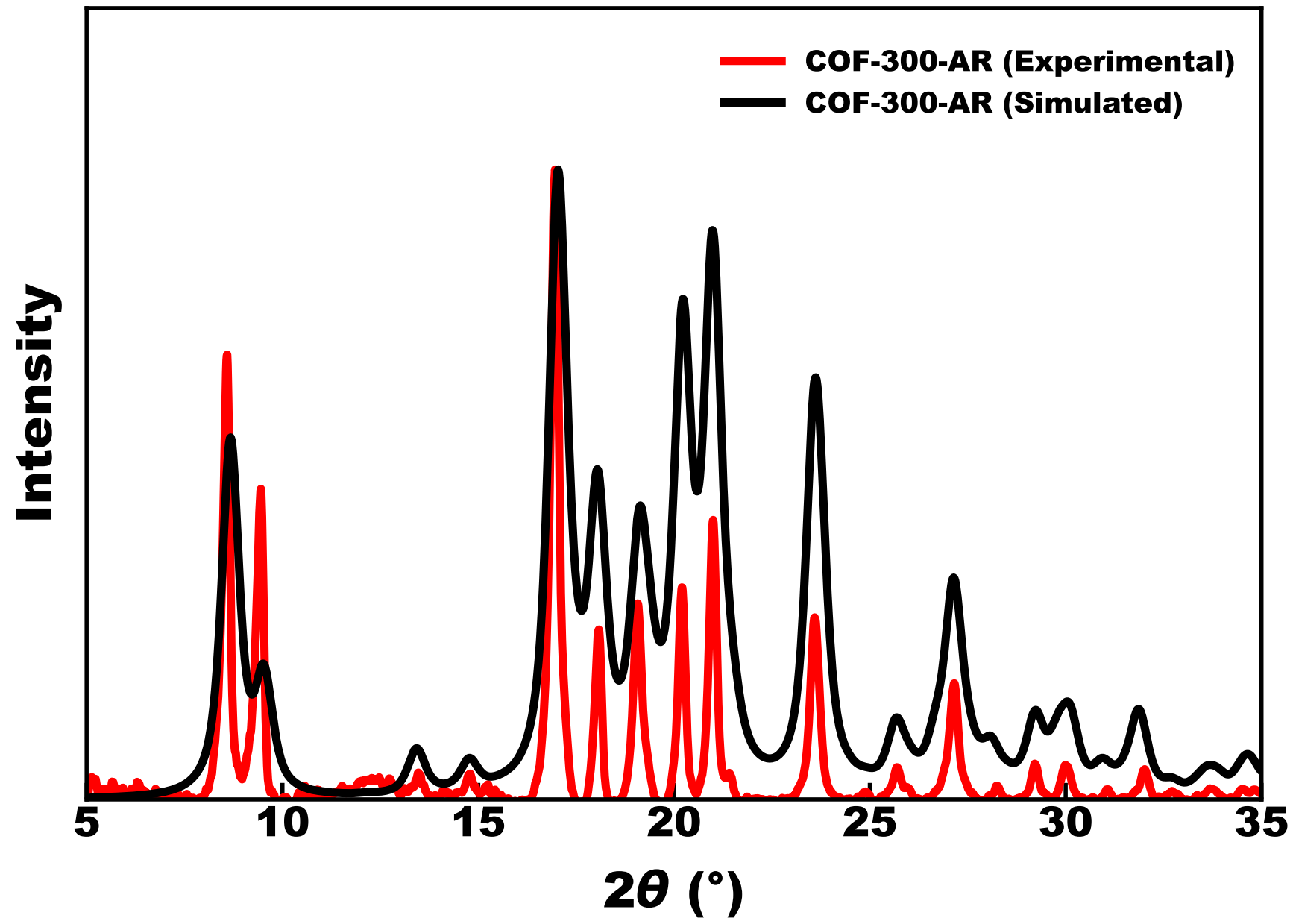

Figure S14: Comparison of experimental (red) and simulated (black) PXRD patterns of COF-300-AR.

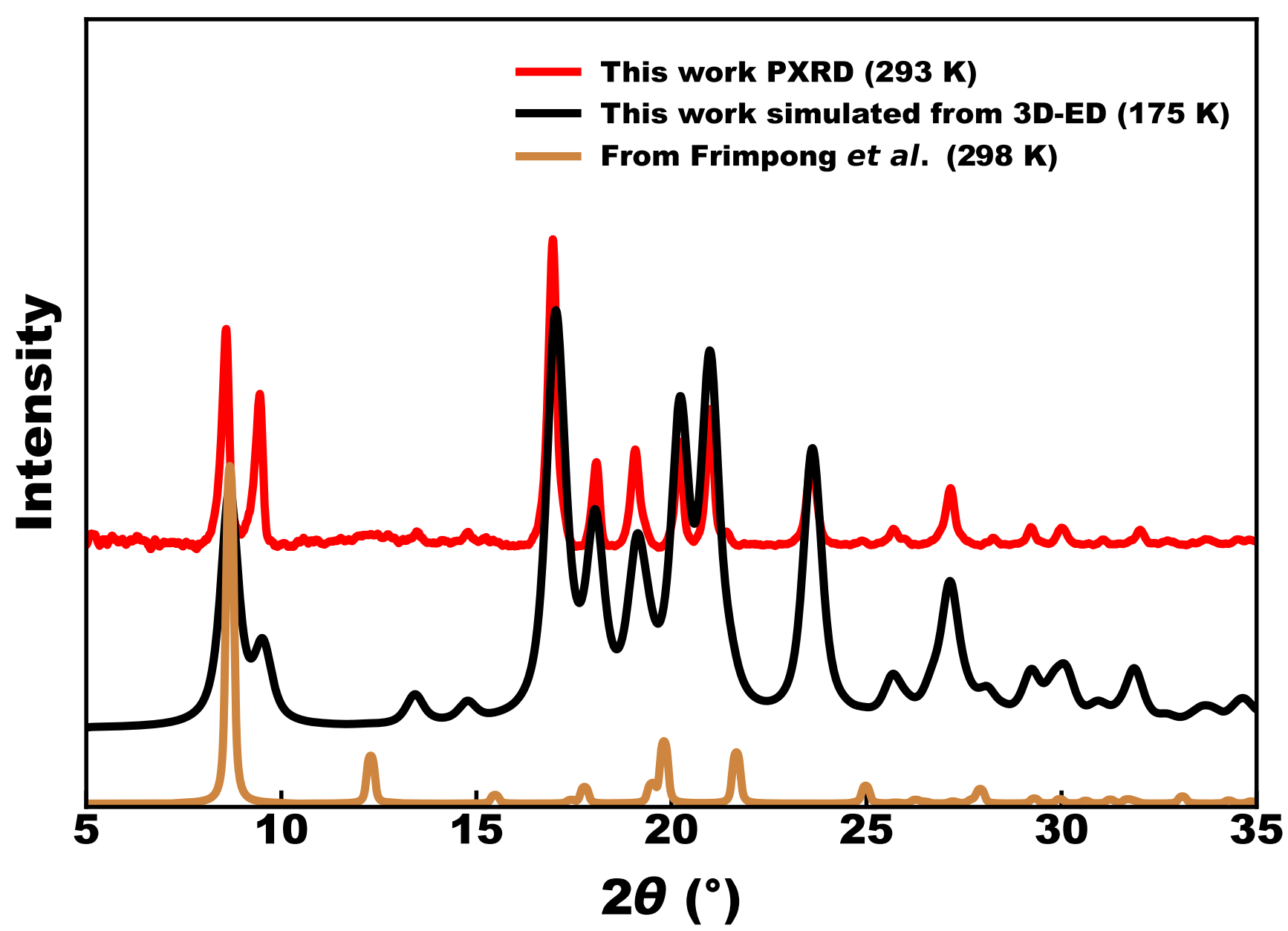

Figure S15: Comparison of PXRD patterns of COF-300-AR phases from this work (red, at 293 K), simulated from the 3D-ED data (this work, black, at 175 K), and as reported by Frimpong *et al.* (brown, at 298 K).[39]

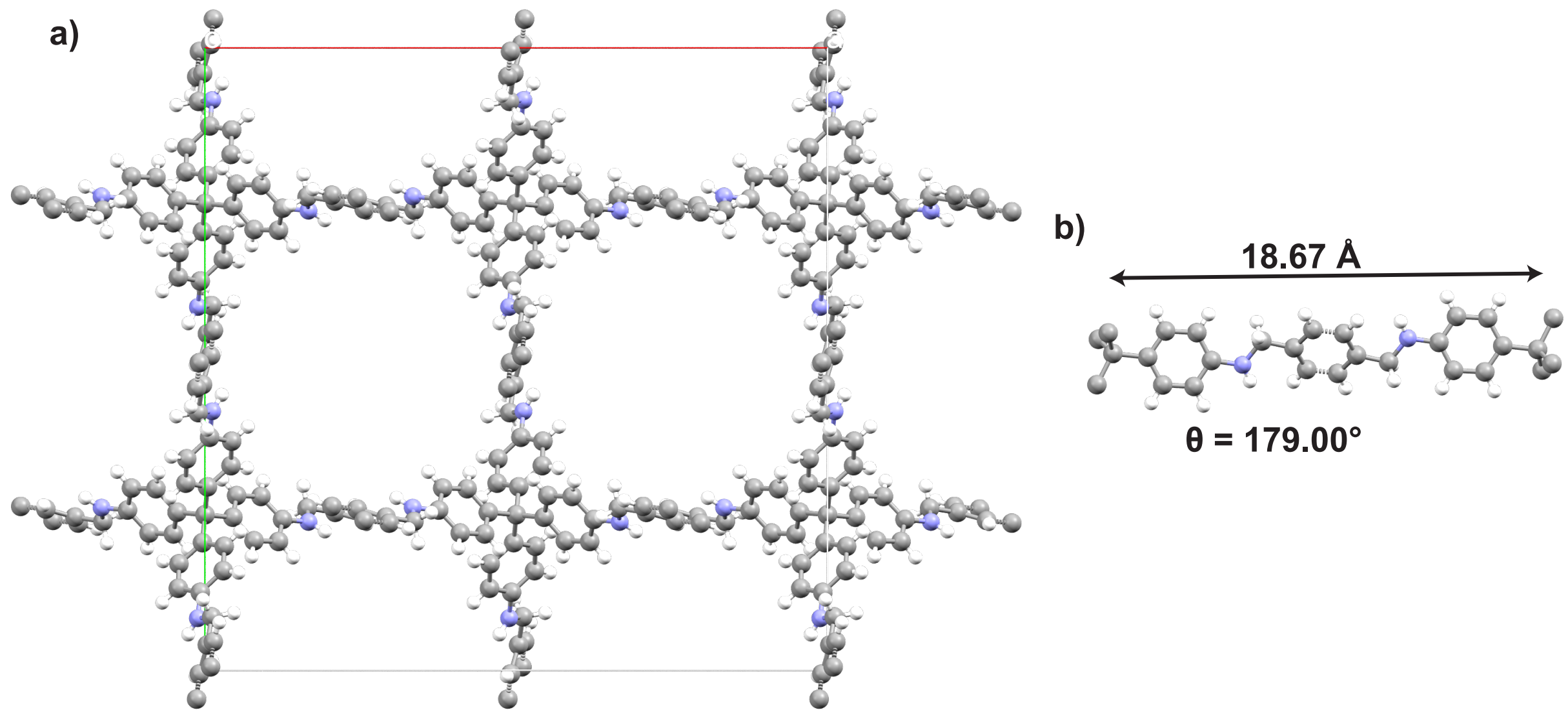

Figure S16: Open-pore COF-300-AR: a) Packing of the unit cell. b) Linker geometry.[2] Carbon atoms are shown in gray, nitrogen atoms in blue, and hydrogen atoms in white.

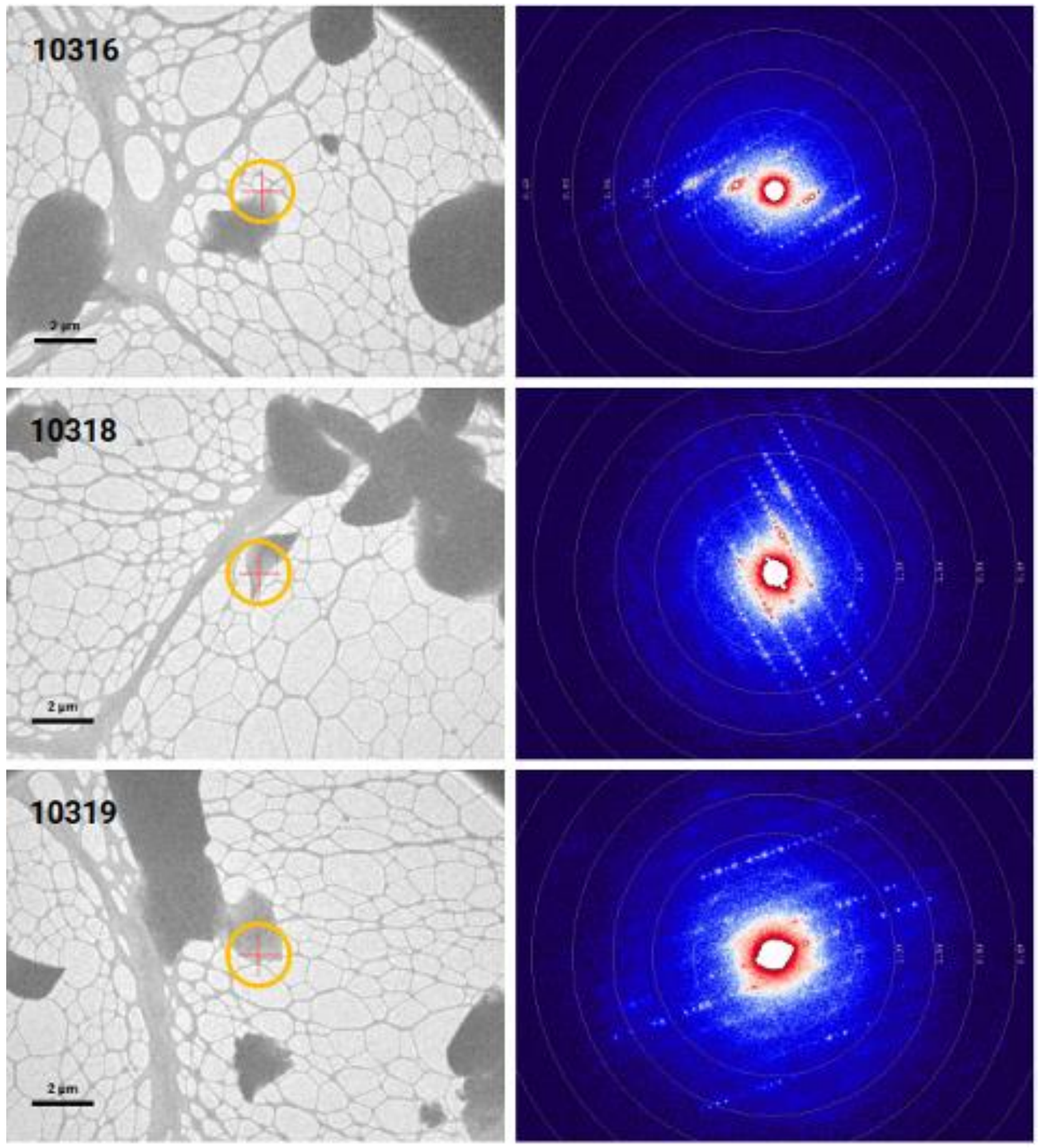

Figure S17: Transmission electron microscope images and exemplary, cropped diffraction patterns for individual measurements of COF-300-AR.

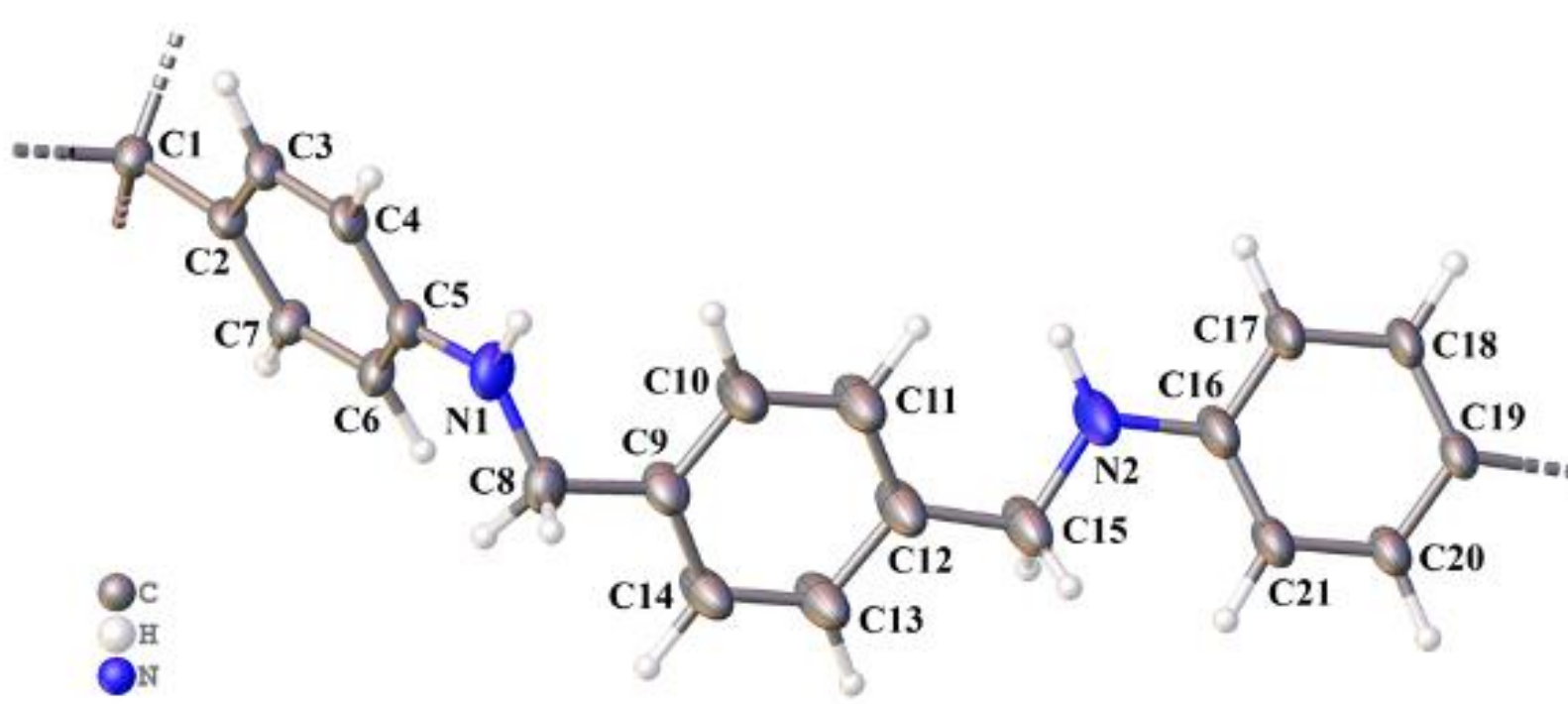

Figure S18: Displacement ellipsoid plot of the asymmetric unit of COF-300-AR obtained from 3D-ED. Displacement ellipsoids are drawn at the 50% probability level.

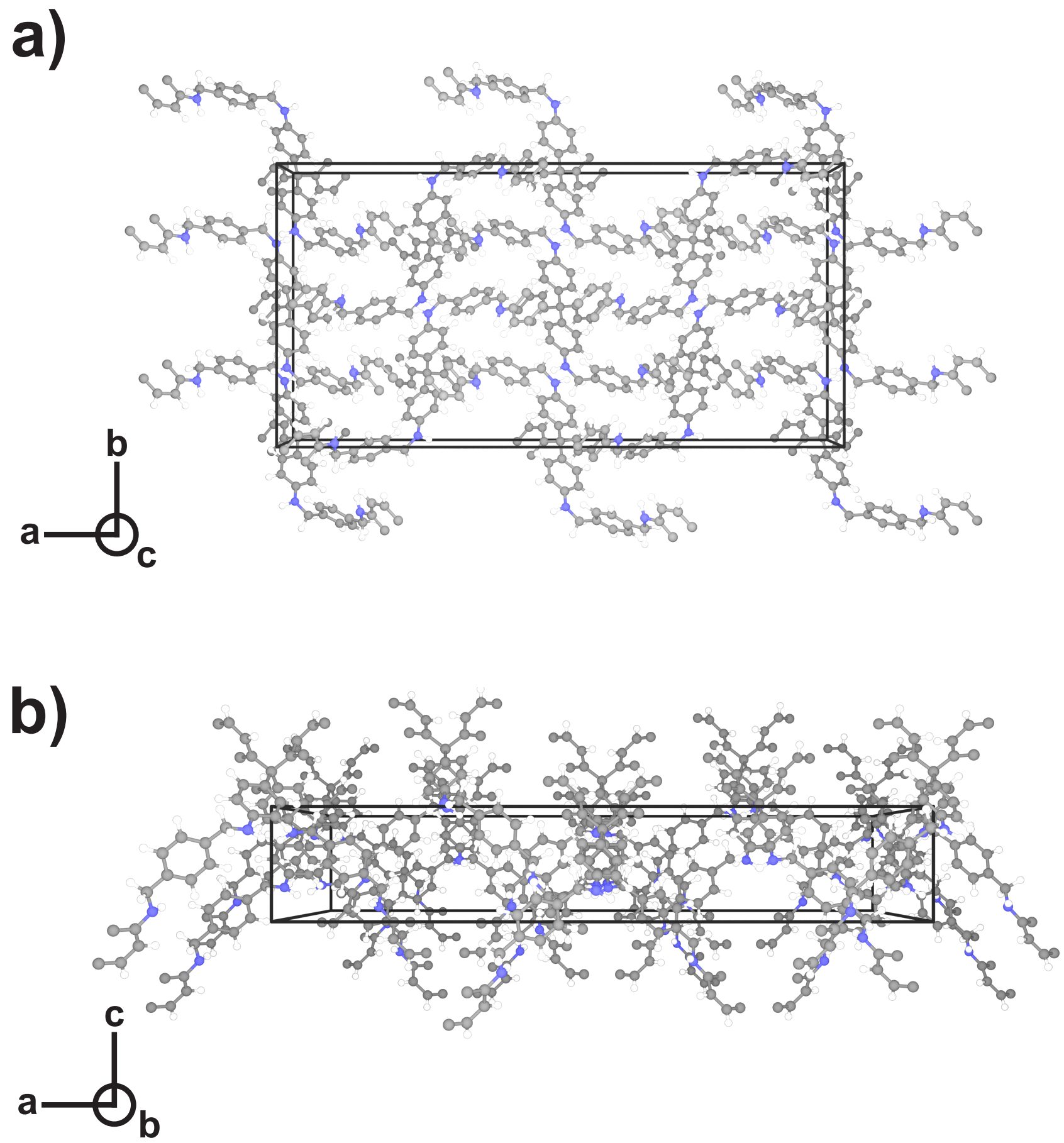

Figure S19: Crystal packing of COF-300-AR viewed along crystallographic *c*-direction (a), and *b*-direction (b). Carbon atoms are shown in gray, nitrogen atoms in blue, and hydrogen atoms in white.

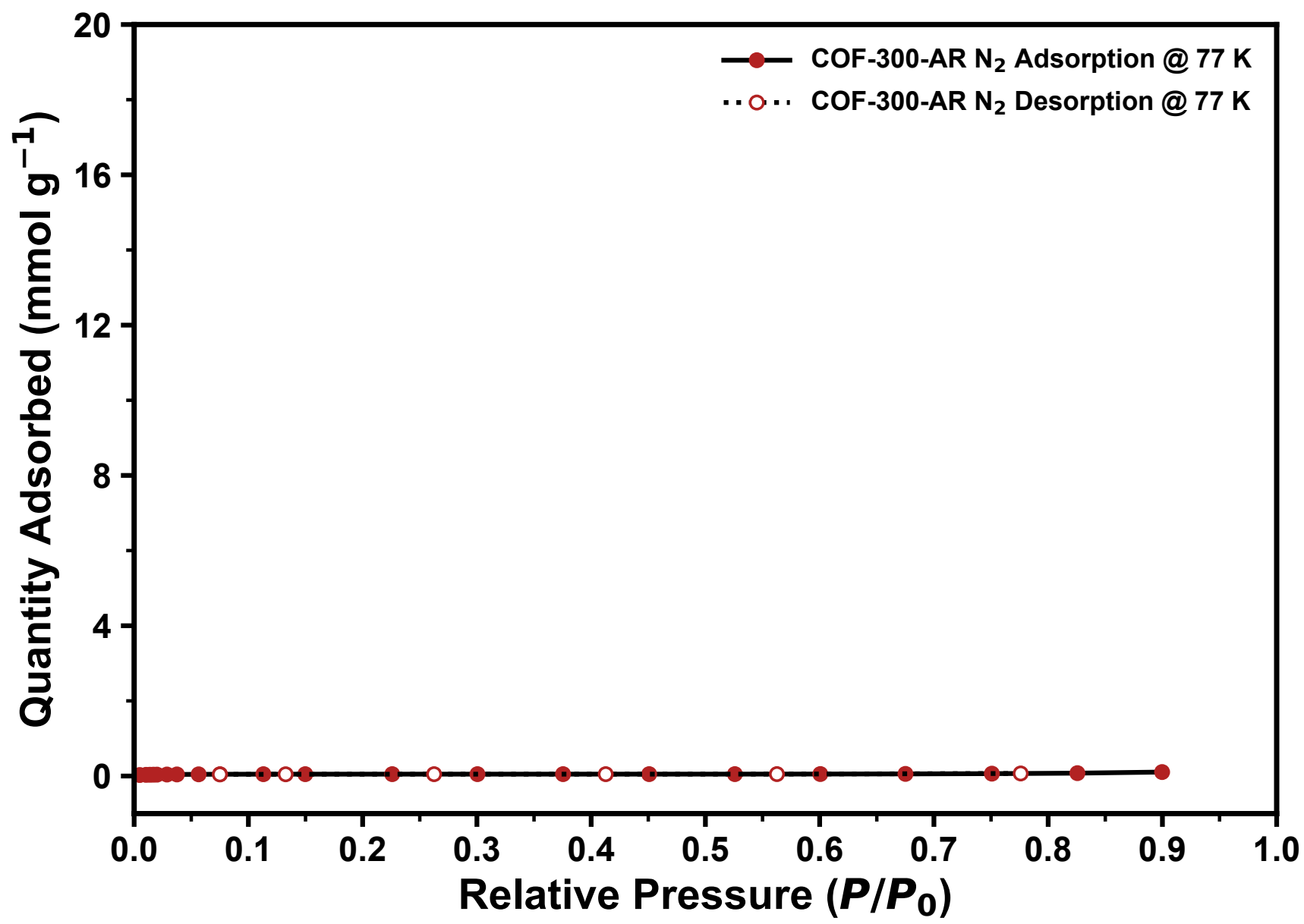

Figure S20: N_2 isotherms of COF-300-AR at 77 K. The closed red data points correspond to adsorption points and the open red points correspond to desorption points.

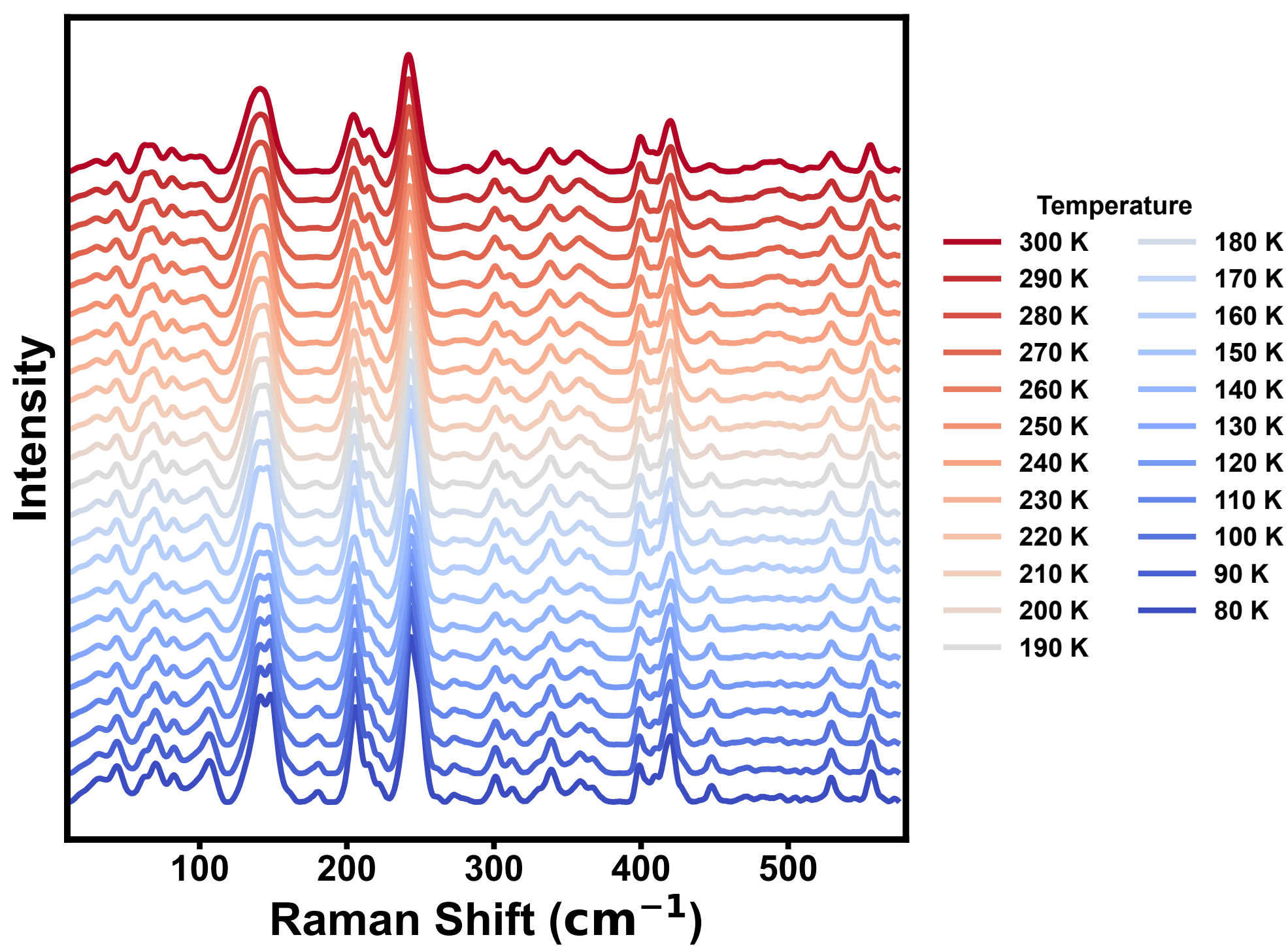

Figure S21: Variable-temperature Raman spectra of COF-300-H_2O.

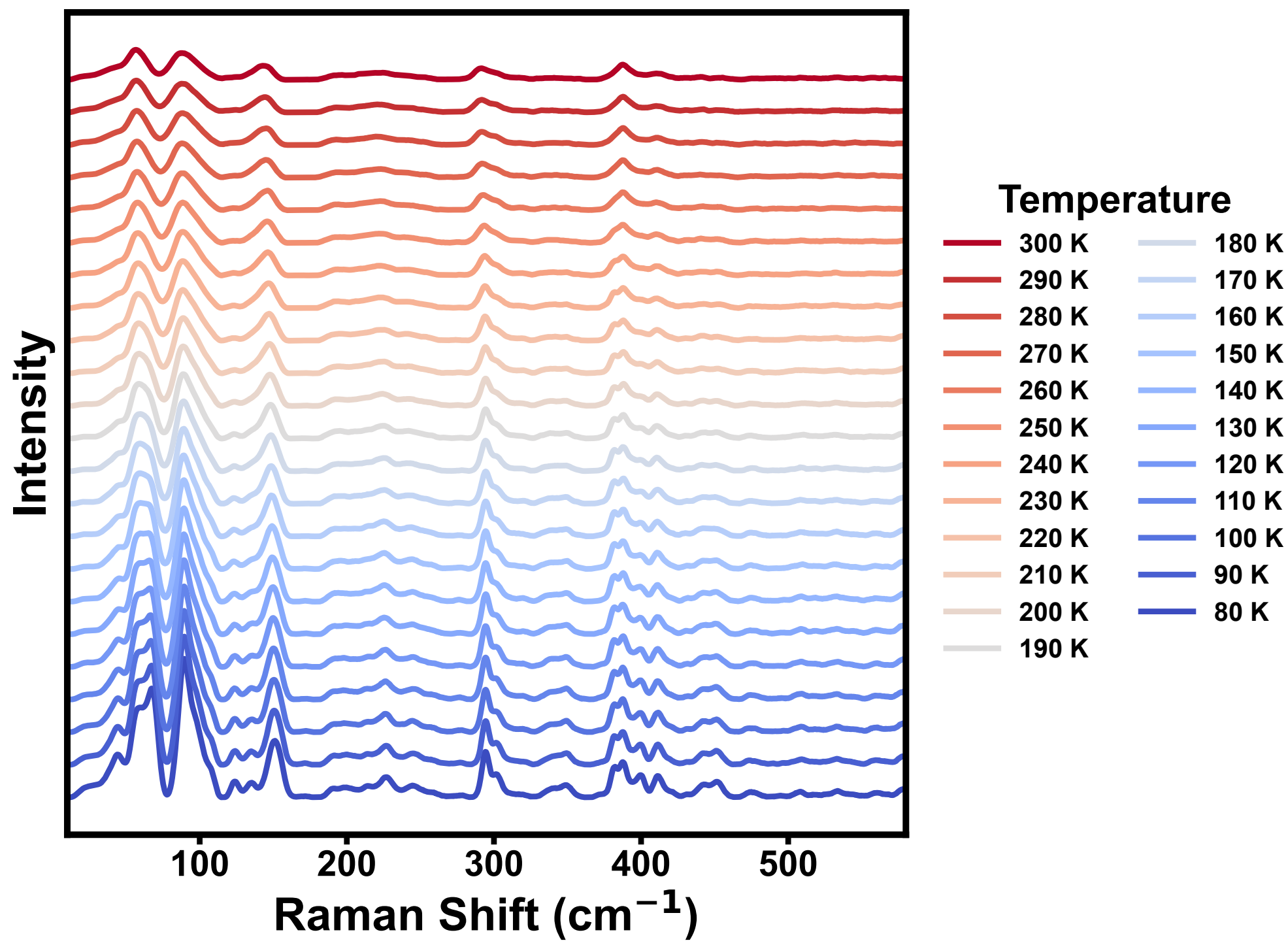

Figure S22: Variable-temperature Raman spectra of COF-300-AR.

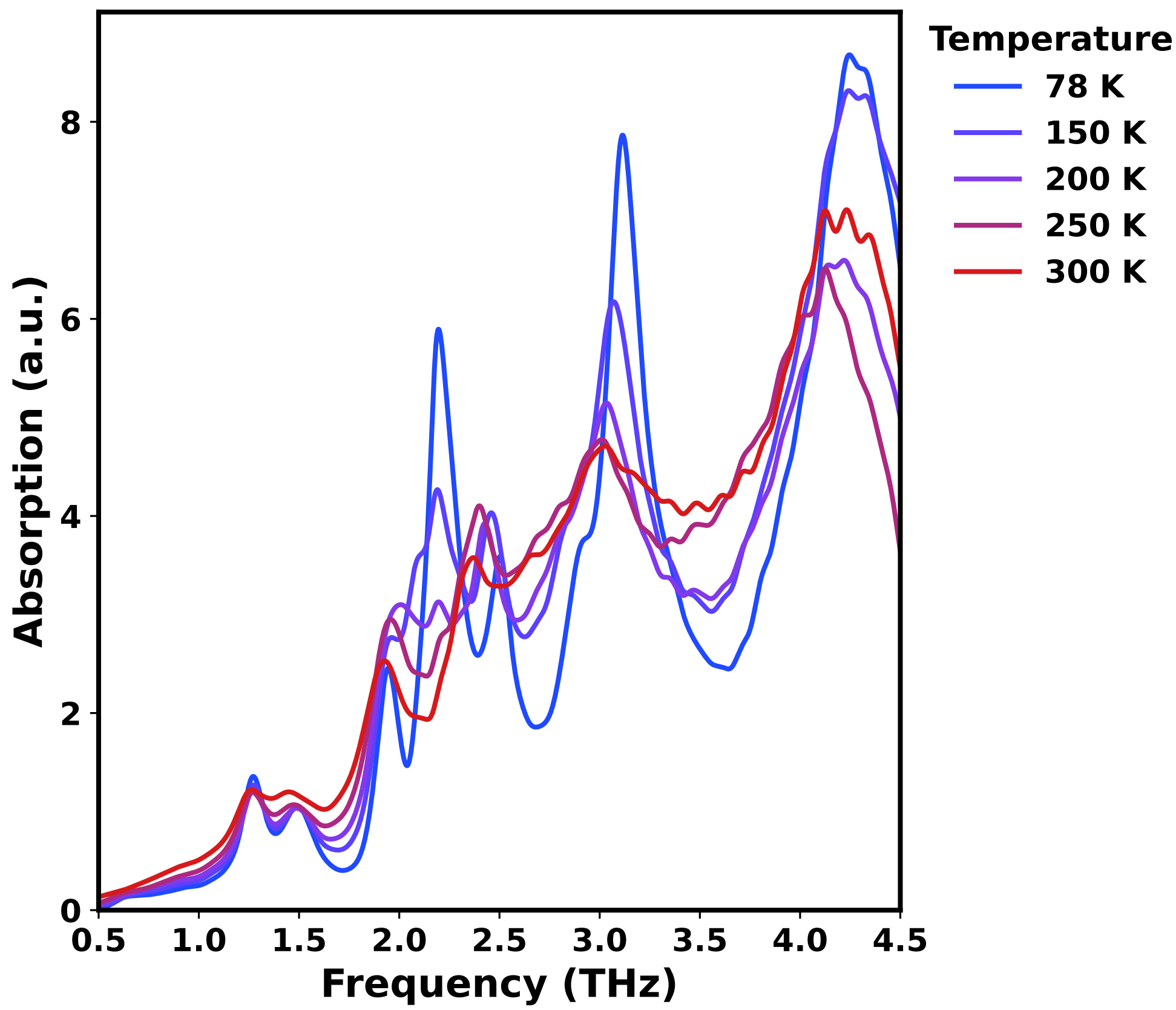

Figure S23: Variable-temperature terahertz spectra of COF-300-H_2O.

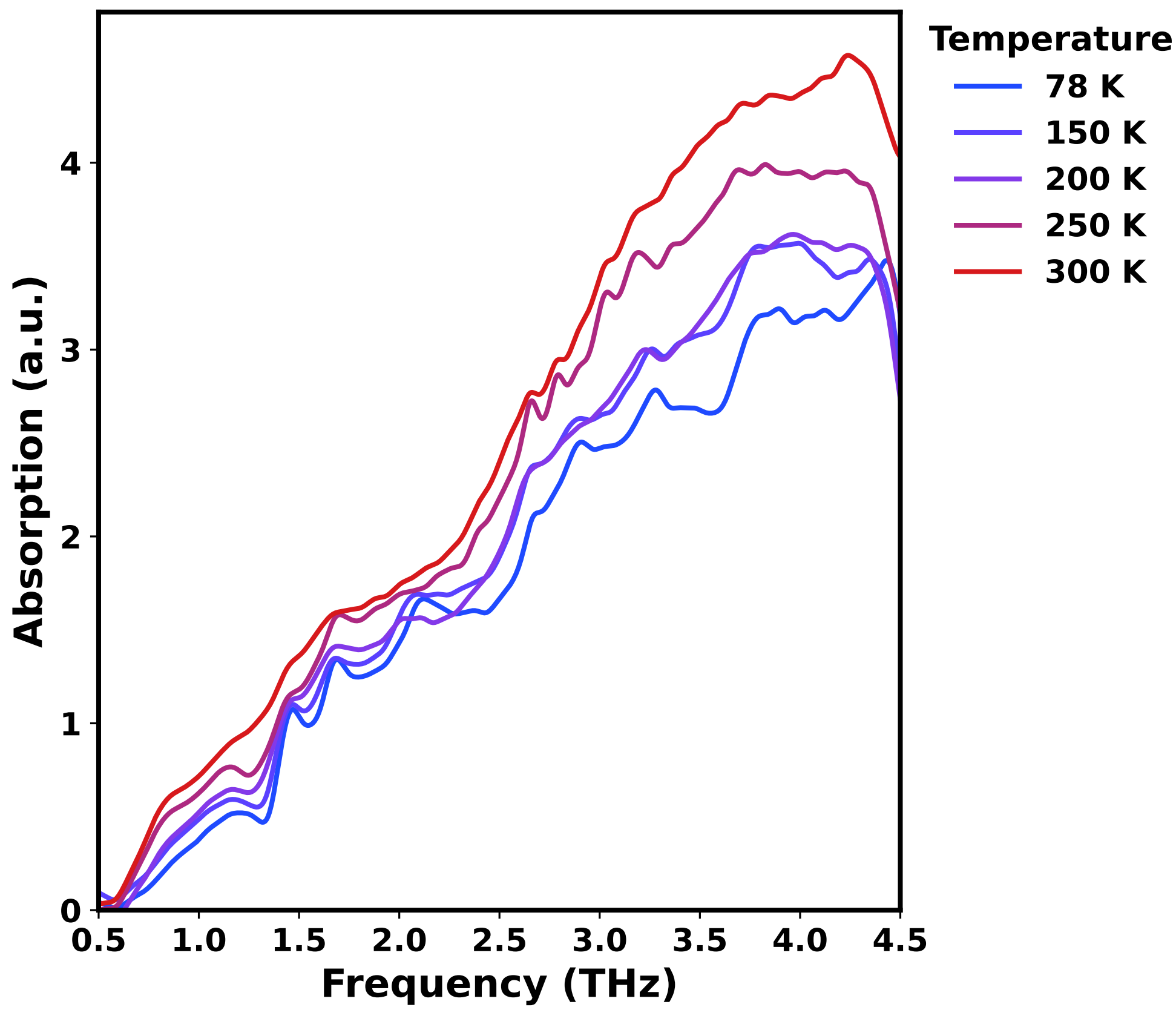

Figure S24: Variable-temperature terahertz spectra of COF-300-AR.

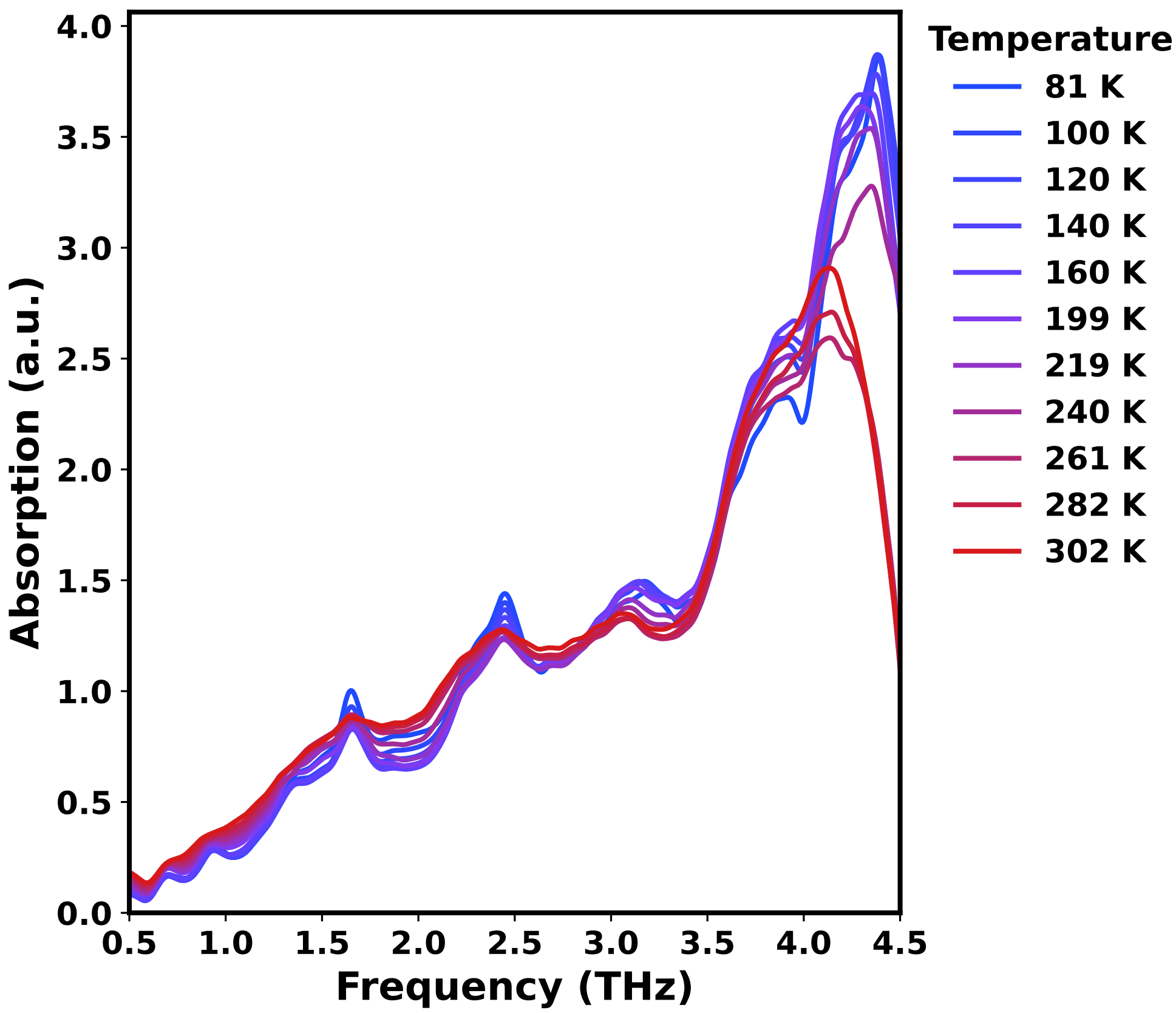

Figure S25: Variable-temperature terahertz spectra of COF-320A.

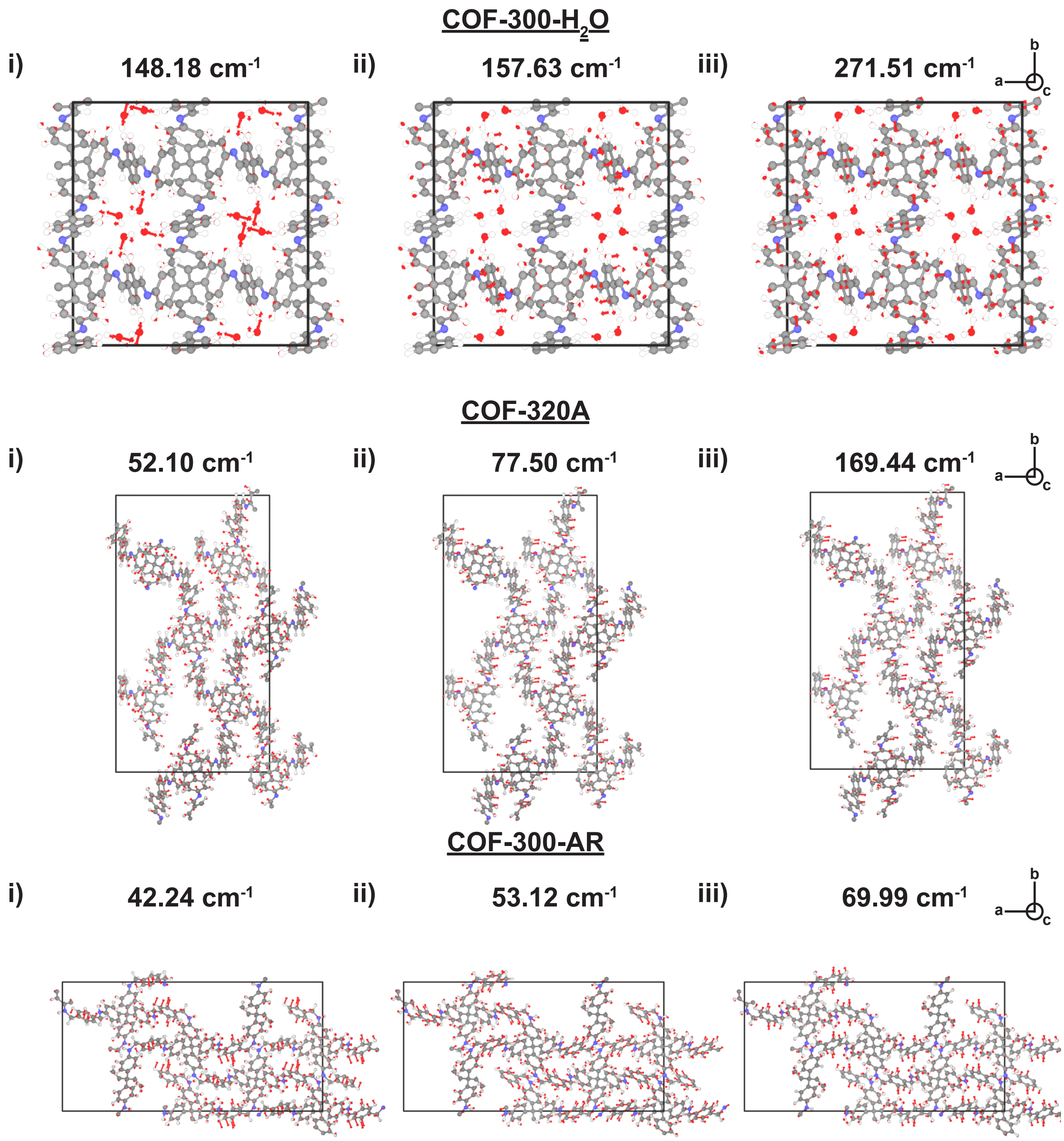

Figure S26: Representative calculated normal-mode displacement patterns for COF-300-H_2O, COF-320A, and COF-300-AR obtained from solid-state DFT vibrational analysis. Red arrows indicate the direction and relative magnitude of the atomic displacements for selected low-frequency modes.

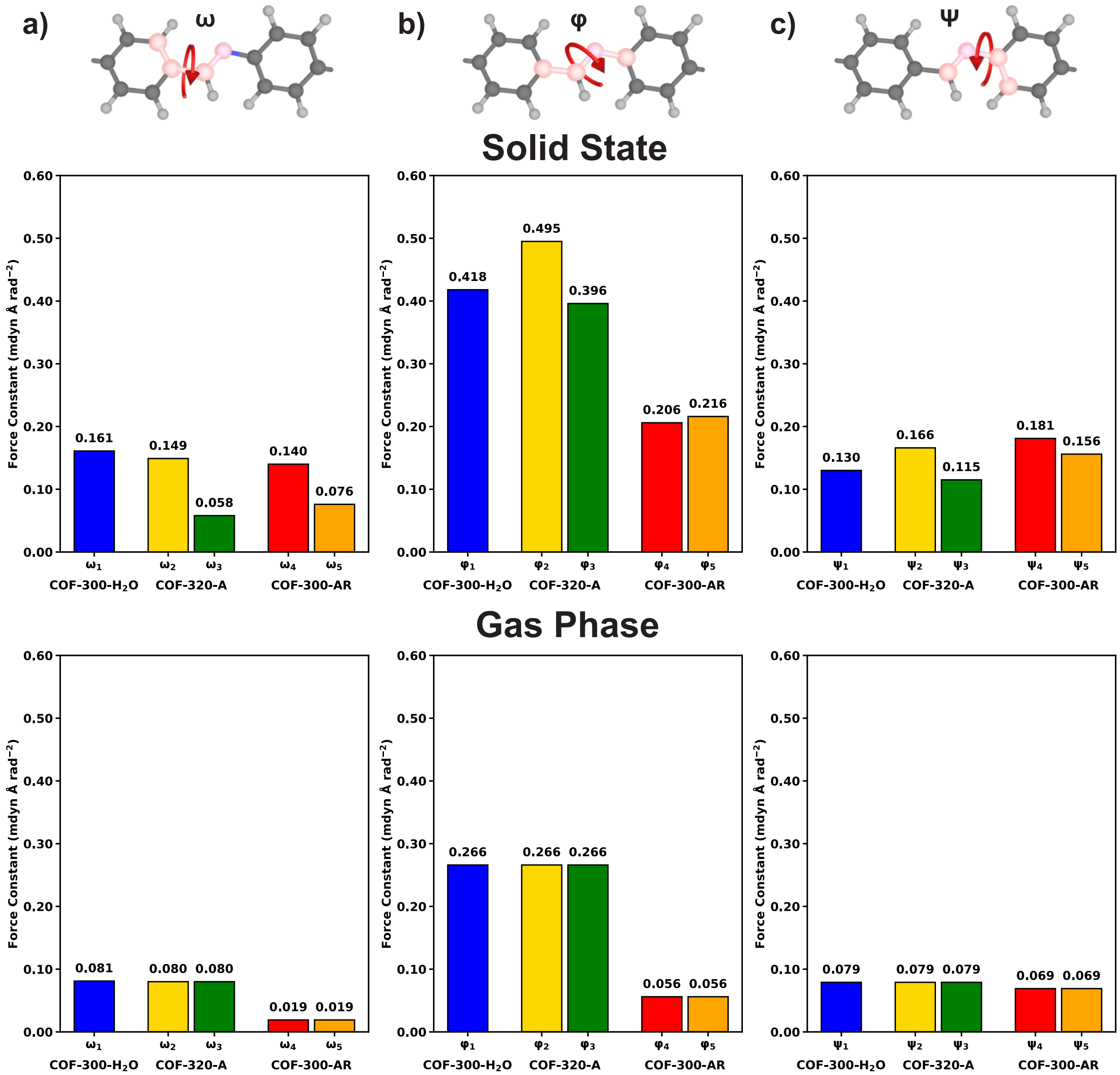

Figure S27: Comparison of the torsional force constants calculated in the solid state (top) and gas phase (bottom) for the pedal motion on the carbon side (a), the bridge (b), and pedal motion on the nitrogen side (c) for COF-300-H_2O, COF-320A, and COF-300-AR.

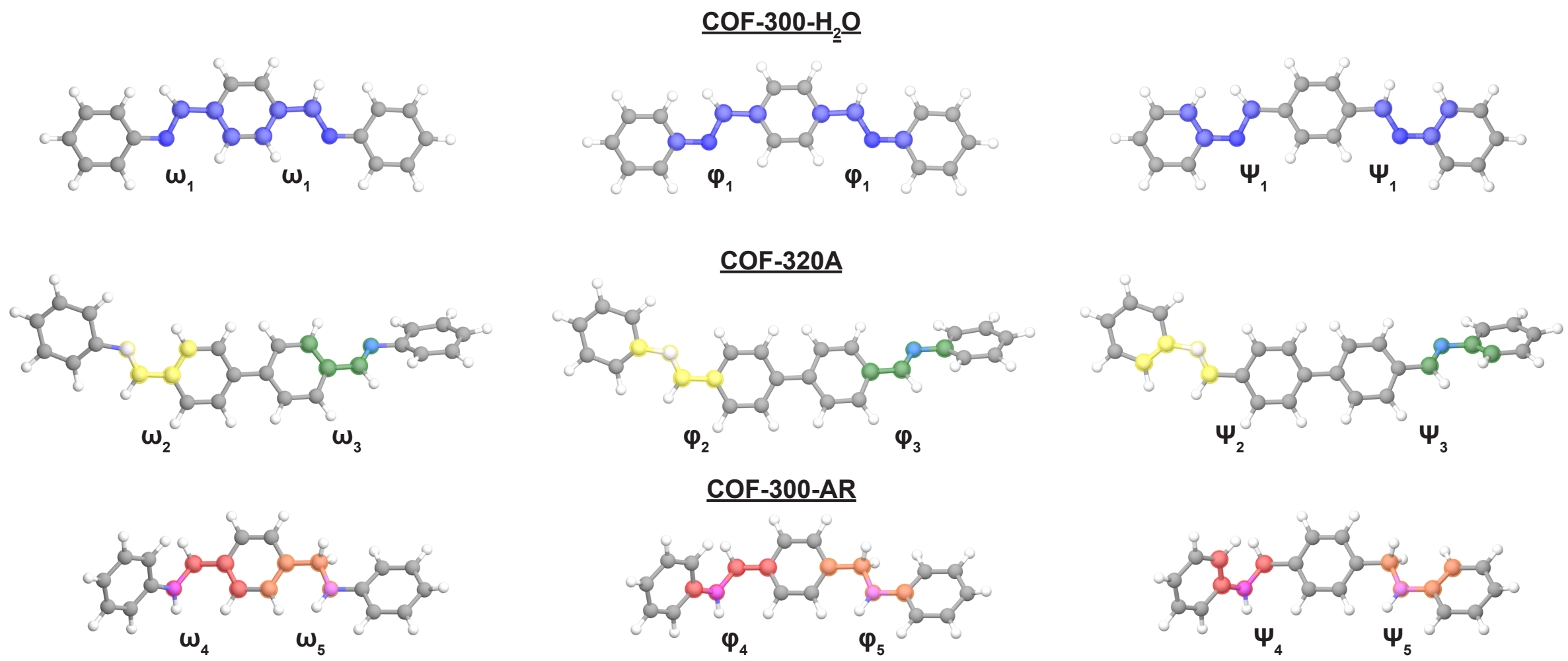

Figure S28: Dihedral angle labels for the representative linker portions used in the gas phase rotational calculations. Note that while in the solid-state a portion of the dihedral angles are symmetry independent, while in the gas phase, the relaxed nature of the scan results in equivalent energy surfaces regardless of which symmetry-related angles are rotated.

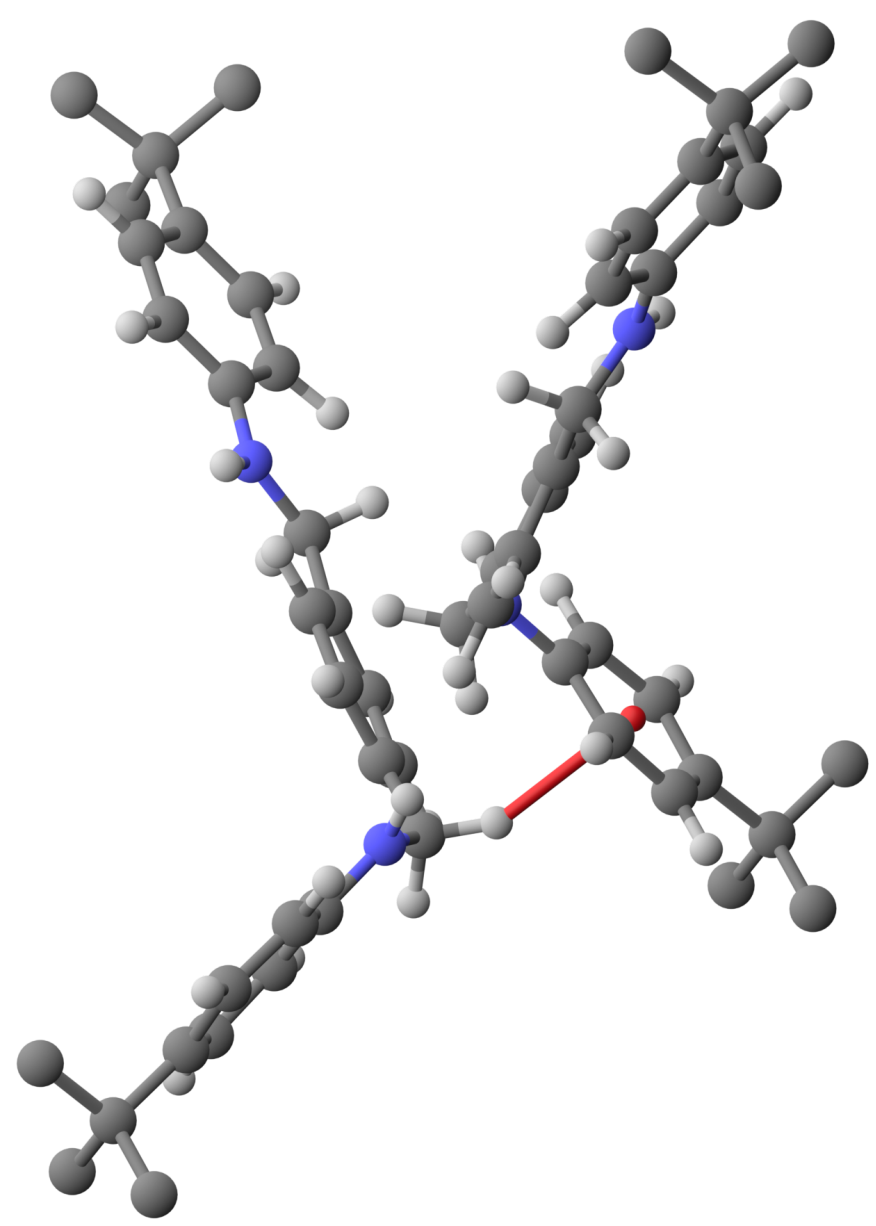

Figure S29: The C–H$\cdots\pi$ interaction between two representative linker portions that stabilizes the bent conformer of contracted COF-300-AR in the solid state. Structure is obtained from 3D-ED. Carbon atoms are shown in gray, nitrogen atoms in blue, and hydrogen atoms in white.

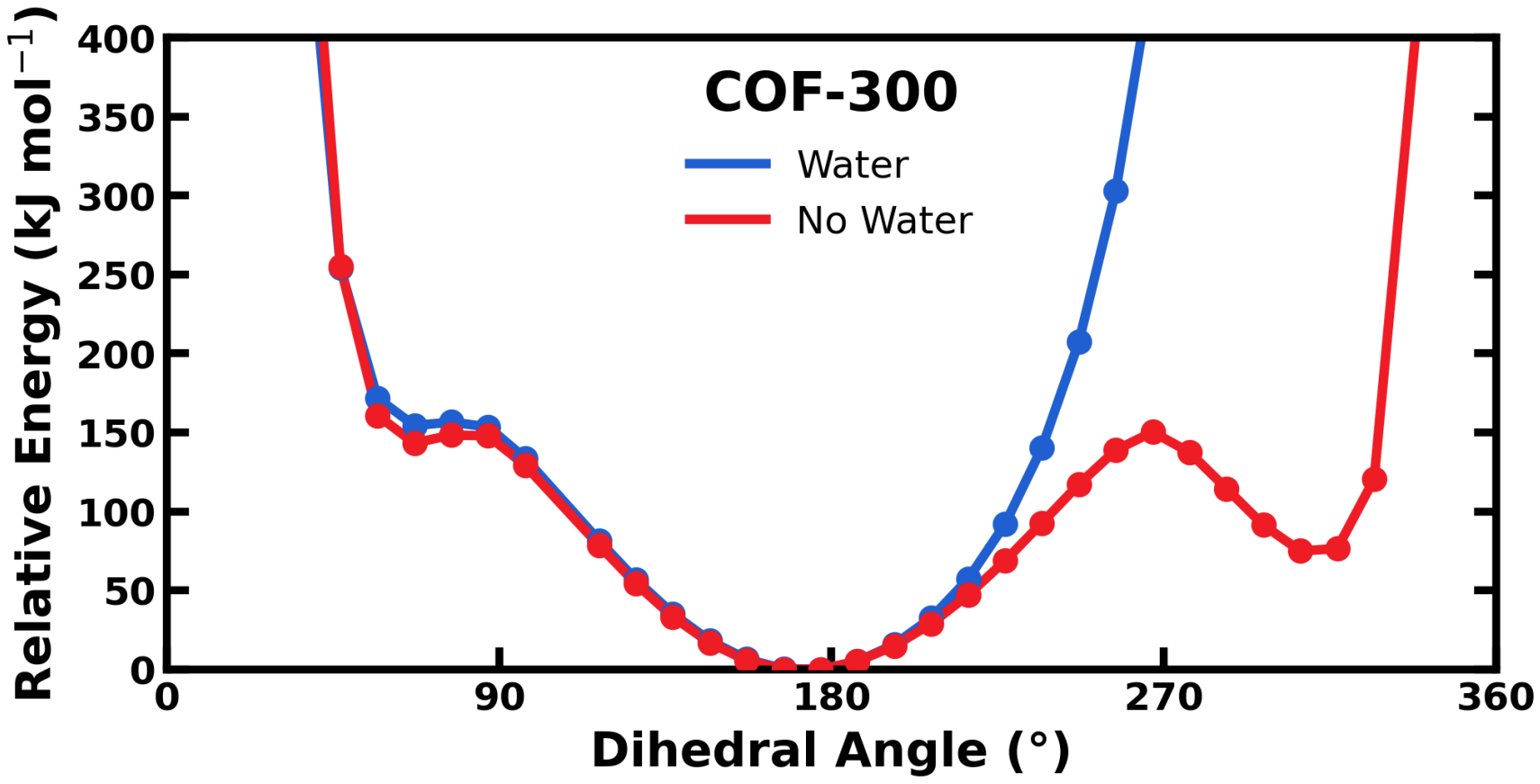

Figure S30: A rigid potential energy surface scan of the imine (N=C) bond in a representative linker fragment of COF-300, with (blue line) and without (red line) a water molecule. The water position was extracted from the crystal structure positioned 2.09 Å from the nitrogen atom in the rotating portion of the molecule.

Mode Visualization

Mode visualizations are included as .xyz files in a compressed folder for COF-300-H_2O, COF-320A, and COF-300-AR.

References

(1) Chen, Y.; Shi, Z.-L.; Wei, L.; Zhou, B.; Tan, J.; Zhou, H.-L.; Zhang, Y.-B. Guest-Dependent Dynamics in a 3D Covalent Organic Framework. *J. Am. Chem. Soc.* **2019**, *141*, 3298–3303.

(2) Li, Y.; Wan, Y.; Deng, X.; Zhao, M.; Zhang, B.; Wang, Z. Postsynthetic Construction of Single-Crystal Formamide-Linked Covalent Organic Frameworks. *J. Am. Chem. Soc.* **2025**, *147*, 29526–29533.

(3) Kang, C.; Zhang, Z.; Kusaka, S.; Negita, K.; Usadi, A. K.; Calabro, D. C.; Baugh, L. S.; Wang, Y.; Zou, X.; Huang, Z.; Matsuda, R. Covalent Organic Framework Atropisomers with Multiple Gas-triggered Structural Flexibilities. *Nat. Mater.* **2023**, *22*, 636–643.

(4) Li, X.; Wang, Q.; Tang, J.; Wu, J.; Ning, Q.; Yu, H.; Huo, J.; He, Y. Hydration-Accelerated Crown Ether Diffusion within Single Three-Dimensional Covalent Organic Frameworks. *Nano Lett.* **2024**, *24*, 9505–9510.

(5) Liu, H.; Chu, J.; Yin, Z.; Cai, X.; Zhuang, L.; Deng, H. Covalent Organic Frameworks Linked by Amine Bonding for Concerted Electrochemical Reduction of CO_2. *Chem* **2018**, *4*, 1696–1709.

(6) Zeng, T.; Ling, Y.; Jiang, W.; Yao, X.; Tao, Y.; Liu, S.; Liu, H.; Yang, T.; Wen, W.; Jiang, S.; Zhao, Y.; Ma, Y.; Zhang, Y.-B. Atomic Observation and Structural Evolution of Covalent Organic Framework Rotamers. *Proc. Natl. Acad. Sci. U. S. A.* **2024**, *121*, e2320237121.

(7) Sun, T.; Wei, L.; Chen, Y.; Ma, Y.; Zhang, Y.-B. Atomic-Level Characterization of Dynamics of a 3D Covalent Organic Framework by Cryo-Electron Diffraction Tomography. *J. Am. Chem. Soc.* **2019**, *141*, 10962–10966.

(8) Zhou, Z.; Cai, G.; Zhang, Z.; Li, G.; Lou, D.; Qu, S.; Li, Y.; Huang, M.; Liu, W.; Zheng, Z.;

others Conformational Chirality of Single-Crystal Covalent Organic Frameworks. *J. Am. Chem. Soc.* **2024**, *146*, 34064–34069.

(9) Liu, S.; Wei, L.; Zeng, T.; Jiang, W.; Qiu, Y.; Yao, X.; Wang, Q.; Zhao, Y.; Zhang, Y.-B. Single-Crystal Dynamic Covalent Organic fFameworks for Adaptive Guest Alignments. *J. Am. Chem. Soc.* **2024**, *146*, 34053–34063.

(10) Yao, A.; Xu, H.; Shao, K.; Sun, C.; Qin, C.; Wang, X.; Su, Z. Guest-Induced Structural Transformation of Single-Crystal 3D Covalent Organic Framework at Room and High Temperatures. *Nat. Commun.* **2025**, *16*, e1385.

(11) Han, J.; Feng, J.; Kang, J.; Chen, J.-M.; Du, X.-Y.; Ding, S.-Y.; Liang, L.; Wang, W. Fast Growth of Single-Crystal Covalent Organic Frameworks for Laboratory X-Ray Diffraction. *Science* **2024**, *383*, 1014–1019.

(12) Gao, W.; Chen, Z.; Hong, J.; Zhang, Y.; Yang, Z.; Liu, M.; Wang, X.; Shang, S.; You, Z.; Shao, Z.; Dong, J. Rapid Synthesis of Single-Crystal Covalent Organic Framework with Controllable Crystal Habits. *J. Am. Chem. Soc.* **2025**, *147*, 15459–15468.

(13) Bourda, L.; Bhandary, S.; Ito, S.; Göb, C. R.; Van Der Voort, P.; Van Hecke, K. Analysis of COF-300 synthesis: Probing Degradation Processes and 3D Electron Diffraction Structure. *IUCrJ* **2024**, *11*, 510–518.

(14) Ito, S.; White, F. J.; Okunishi, E.; Aoyama, Y.; Yamano, A.; Sato, H.; Ferrara, J. D.; Jasnowski, M.; Meyer, M. Structure Determination of Small Molecule Compounds by an Electron Diffractometer for 3D ED/MicroED. *CrystEngComm* **2021**, *23*, 8622–8630.

(15) Truong, K.-N.; Ito, S.; Wojciechowski, J. M.; Göb, C. R.; Schürmann, C. J.; Yamano, A.; Del Campo, M.; Okunishi, E.; Aoyama, Y.; Mihira, T.; Hosogi, N. Making the Most of 3D Electron Diffraction: Best Practices to Handle a New Tool. *Symmetry* **2023**, *15*, e1555.

(16) Rigaku Oxford Diffraction CrysAlisPro Software System. 2025; Software.

(17) Sheldrick, G. M. SHELXT–Integrated Space-Group and Crystal-Structure Determination. *Acta Crystallogr. A* **2015**, *71*, 3–8.

(18) Sheldrick, G. M. Crystal Structure Refinement with SHELXL. *Acta Crystallogr. C* **2015**, *71*, 3–8.

(19) Dolomanov, O. V.; Bourhis, L. J.; Gildea, R. J.; Howard, J. A.; Puschmann, H. OLEX2: A Complete Structure Solution, Refinement and Analysis Program. *J. Appl. Crystallogr.* **2009**, *42*, 339–341.

(20) Rigaku Oxford Diffraction AutoChem 7 Software System in Conjunction with OLEX2. 2025; Software.

(21) Hirshfeld, F. Can X-ray Data Distinguish Bonding Effects from Vibrational Smearing? *Acta Crystallogr. A* **1976**, *32*, 239–244.

(22) Thorn, A.; Dittrich, B.; Sheldrick, G. M. Enhanced Rigid-Bond Restraints. *Acta Crystallogr. A* **2012**, *68*, 448–451.

(23) Stoll, W. B.; Banks, P. A.; Dannenberg, S. G.; Waterman, R.; Catalano, L.; Ruggiero, M. T. Interrogation of the Intermolecular Forces That Drive Bulk Properties of Molecular Crystals with Terahertz Spectroscopy and Density Functional Theory. *Cryst. Growth Des.* **2025**, *25*, 3697–3706.

(24) Erba, A.; Desmarais, J. K.; Casassa, S.; Civalleri, B.; Donà, L.; Bush, I. J.; Searle, B.; Maschio, L.; Edith-Daga, L.; Cossard, A.; Ribaldone, C. CRYSTAL23: A Program for Computational Solid State Physics and Chemistry. *J. Chem. Theory Comput.* **2022**, *19*, 6891–6932.

(25) Schiafer, A.; Horn, H.; Ahlrichs, R. Fully Optimized Contracted Gaussian Basis Sets for Atoms Li to Kr. *J. Chem. Phys* **1992**, *97*, 2571.

(26) Perdew, J. P.; Burke, K.; Ernzerhof, M. Generalized Gradient Approximation Made Simple. *Phys. Rev. Lett.* **1996**, *77*, 3865–3368.

(27) Grimme, S.; Ehrlich, S.; Goerigk, L. Effect of the Damping Function in Dispersion Corrected Density Functional Theory. *J. Comput. Chem.* **2011**, *32*, 1456–1465.

(28) Grimme, S.; Antony, J.; Ehrlich, S.; Krieg, H. A Consistent and Accurate ab initio Parametrization of Density Functional Dispersion Correction (DFT-D) for the 94 Elements H-Pu. *J. Chem. Phys.* **2010**, *132*, e154104.

(29) Civalleri, B.; D'Arco, P.; Orlando, R.; Saunders, V.; Dovesi, R. Hartree–Fock Geometry Optimisation of Periodic Systems with the CRYSTAL Code. *Chem. Phys. Lett.* **2001**, *348*, 131–138.

(30) Pascale, F.; Zicovich-Wilson, C. M.; López Gejo, F.; Civalleri, B.; Orlando, R.; Dovesi, R. The Calculation of the Vibrational Frequencies of Crystalline Compounds and Its Implementation in the CRYSTAL Code. *J. Comp. Chem.* **2004**, *25*, 888–897.

(31) Zicovich-Wilson, C. M.; Pascale, F.; Roetti, C.; Saunders, V. R.; Orlando, R.; Dovesi, R. Calculation of the Vibration Frequencies of α-Quartz: The Effect of Hamiltonian and Basis Set. *J. Comp. Chem.* **2004**, *25*, 1873–1881.

(32) Noel, Y.; Zicovich-Wilson, C. M.; Civalleri, B.; D'Arco, P.; Dovesi, R. Polarization Properties of ZnO and BeO: An ab initio Study Through the Berry Phase and Wannier Functions Approaches. *Phys. Rev. B* **2001**, *65*, e014111.

(33) Maschio, L.; Kirtman, B.; Rérat, M.; Orlando, R.; Dovesi, R. Ab initio Analytical Raman Intensities for Periodic Systems Through a Coupled Perturbed Hartree-Fock/Kohn-Sham Method in an Atomic Orbital Basis. I. Theory. *J. Chem. Phys.* **2013**, *139*, 164101.

(34) Kraka, E.; Zou, W.; Tao, Y. Decoding Chemical Information from Vibrational Spectroscopy

Data: Local Vibrational Mode Theory. *Wiley Interdiscip. Rev. Comput. Mol. Sci.* **2020**, *10*, e1480.

(35) Bodo, F.; Erba, A.; Kraka, E.; Moura Jr, R. T. Chemical Bonding in Uranium-Based Materials: A Local Vibrational Mode Case Study of $Cs_2UO_2Cl_4$ and UCl_4 Crystals. *J. Comput. Chem.* **2024**, *45*, 1130–1142.

(36) Frisch, M. J. et al. Gaussian16 Revision C.01. 2016; Gaussian Inc. Wallingford CT.

(37) Konkoli, Z.; Cremer, D. A New Way of Analyzing Vibrational Spectra. I. Derivation of Adiabatic Internal Modes. *Int. J. Quantum Chem.* **1998**, *67*, 1–9.

(38) Zou, W.; Cremer, D. *Thom H. Dunning, Jr. A Festschrift from Theoretical Chemistry Accounts*; Springer, 2014; pp 149–163.

(39) Frimpong, S. O.; Pacheco, M.; Xie, H.; Zeppuhar, A. N.; Farha, O. K.; Taylor, M. K. Tailoring a 3D Covalent Organic Framework Toward Facile Functionalization. *Small* **2025**, *22*, e11087.
